# Stress-Induced Translation Inhibition through Rapid Displacement of Scanning Initiation Factors

**DOI:** 10.1016/j.molcel.2020.09.021

**Published:** 2020-11-05

**Authors:** Stefan Bresson, Vadim Shchepachev, Christos Spanos, Tomasz W. Turowski, Juri Rappsilber, David Tollervey

**Affiliations:** 1 Wellcome Centre for Cell Biology, University of Edinburgh, Edinburgh, UK; 2 Bioanalytics, Institute of Biotechnology, Technische Universität Berlin, 13355 Berlin, Germany

**Keywords:** protein-RNA interaction, RNA-binding sites, UV crosslinking, mass spectrometry, yeast, stress responses

## Abstract

Cellular responses to environmental stress are frequently mediated by RNA-binding proteins (RBPs). Here, we examined global RBP dynamics in *Saccharomyces cerevisiae* in response to glucose starvation and heat shock. Each stress induced rapid remodeling of the RNA-protein interactome without corresponding changes in RBP abundance. Consistent with general translation shutdown, ribosomal proteins contacting the mRNA showed decreased RNA association. Among translation components, RNA association was most reduced for initiation factors involved in 40S scanning (eukaryotic initiation factor 4A [eIF4A], eIF4B, and Ded1), indicating a common mechanism of translational repression. In unstressed cells, eIF4A, eIF4B, and Ded1 primarily targeted the 5′ ends of mRNAs. Following glucose withdrawal, 5′ binding was abolished within 30 s, explaining the rapid translation shutdown, but mRNAs remained stable. Heat shock induced progressive loss of 5′ RNA binding by initiation factors over ∼16 min and provoked mRNA degradation, particularly for translation-related factors, mediated by Xrn1. Taken together, these results reveal mechanisms underlying translational control of gene expression during stress.

## Highlights

• A quantitative proteomic approach reveals global stress-induced changes in RNA binding
• Translation shutdown is driven by rapid loss of mRNA binding by key initiation factors
• Heat shock induces general mRNA degradation facilitated by Xrn1

## Graphical Abstract

**Figure:**
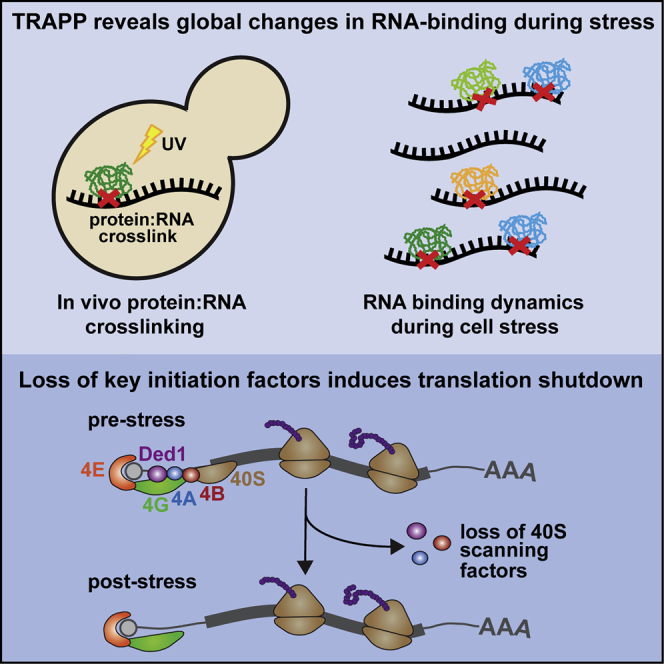

## Teaser

All organisms live in continuously changing environments, and RNA-binding proteins are critically important in responding to resulting stresses. Bresson et al. followed both global and specific changes in RNA-protein interactions over short times following glucose starvation or heat shock. The results reveal mechanisms underlying translational control of gene expression during stress.

## Introduction

All organisms are subject to a continuously changing environment, to which they must adapt in order to survive. This problem is especially acute for unicellular, non-motile organisms, such as the budding yeast *Saccharomyces cerevisiae*. In general, budding yeast respond to stress by inducing global changes in gene expression. At the transcriptional level, this involves the activation of the environmental stress response, in which hundreds of stress-response genes are upregulated and genes encoding ribosome maturation and protein synthesis factors are suppressed. To a large extent, these coordinated changes in gene expression are induced regardless of the identity of the initiating stress (Gasch et al., 2000).

Transcriptional reprogramming is complemented with rapid posttranscriptional changes, particularly at the level of protein synthesis. Cytoplasmic translation is dramatically attenuated in response to a variety of environmental stresses, including various types of nutrient deprivation, but also physical stresses involving changes in temperature, osmotic balance, or oxidation state. In terms of both speed and scale, glucose starvation triggers the most drastic translational shutdown of any stress (Ashe et al., 2000; Kuhn et al., 2001). Glucose is the preferred energy and carbon source for yeast, and its absence quickly reduces cellular biosynthetic capacity. Physical stresses, such as heat shock, can similarly limit biosynthesis while also damaging the existing proteome. In response, cells halt bulk protein synthesis until protective measures are in place.

Translation initiation is generally the rate-limiting step in protein synthesis and a frequent target of regulation, including during stress (reviewed in Crawford and Pavitt, 2019; Dever et al., 2016; and Janapala et al., 2019). The initiation process begins with assembly of the 43S preinitiation complex (PIC), composed of the 40S subunit plus eukaryotic initiation factor 1 (eIF1), eIF1A, eIF3, eIF5, and eIF2:GTP in complex with the initiator tRNA (Hinnebusch, 2017). In parallel, the mRNA is activated for translation by the eIF4F complex, consisting of the cap-binding protein eIF4E, the scaffolding subunit eIF4G, and the ATP-dependent helicases eIF4A and Ded1 (Gao et al., 2016). The PIC is recruited to the 5′ end of the mRNA with the help of the eIF4F complex, eIF3, and eIF4B (Mitchell et al., 2010; Park et al., 2011; Sen et al., 2015; Walker et al., 2013) and begins scanning along the transcript until it reaches the start codon. The scanning process is aided by the helicase activities of Ded1 and eIF4A, which help unwind secondary structure ahead of the translocating ribosome. Ded1 is preferentially required for translation of mRNAs with highly structured 5′ UTRs, whereas eIF4A is required for optimal translation of all mRNAs, regardless of secondary structure (Iserman et al., 2020; Sen et al., 2015). eIF4B may also assist in the scanning process through its stimulatory effect on eIF4A (Andreou et al., 2017; Sen et al., 2015, 2016; Walker et al., 2013).

The best-studied example of translational control during stress involves the heterotrimeric eIF2 complex, which delivers the initiator tRNA to the 43S PIC. Amino acid starvation triggers the activation of the Gcn2 kinase, whose sole target is a conserved serine residue of eIF2α (Dever et al., 1992; Dey et al., 2005; Harding et al., 2000). Phosphorylated eIF2α blocks recycling of the complex for use in subsequent rounds of translation and thus impairs bulk translation initiation. The Gcn2-eIF2α pathway is highly conserved throughout eukaryotes (Castilho et al., 2014), but, at least in yeast, it is activated in only a limited number of stress conditions (reviewed in Simpson and Ashe, 2012). Notably, eIF2α phosphorylation is not required for translation inhibition in response to glucose withdrawal (Ashe et al., 2000) or heat shock (Grousl et al., 2009). The mechanism of translational arrest during these stresses remains unknown.

Historically, a powerful tool for analyzing protein-RNA interactions, including those involved in translation, has been ultraviolet (UV) crosslinking (Dreyfuss et al., 1984). *In vivo* irradiation with UV light induces covalent crosslinks between protein and RNA. Subsequently, specific purification of a given protein allows for the identification of bound RNAs (the crosslinking and analysis of cDNA [CRAC] and CLIP methods). In a reciprocal approach, the RNA itself can be used to pull down associated proteins. Polyadenylated transcripts can be purified using oligo(dT) selection (Castello et al., 2012; Dreyfuss et al., 1984, 1993; Perez-Perri et al., 2018), but this approach is not applicable to the majority of eukaryotic RNAs, including immature mRNAs, rRNA, tRNA, and a host of additional noncoding RNAs. More recently, alternative methods have been developed to purify RNA, regardless of class (Bao et al., 2018; Huang et al., 2018; Queiroz et al., 2019; Shchepachev et al., 2019; Trendel et al., 2019; Urdaneta et al., 2019). One such technique is TRAPP (total RNA-associated proteome purification), which takes advantage of the intrinsic affinity between RNA and silica to isolate crosslinked protein-RNA complexes (Shchepachev et al., 2019).

Here, we applied TRAPP to yeast cells exposed to either glucose withdrawal or heat shock. This revealed extensive remodeling of the yeast protein-RNA interactome in response to stress and provides insights into the mechanism of translational repression.

## Results

### Global RBP Dynamics in Response to Cell Stress

**Figure 1:**
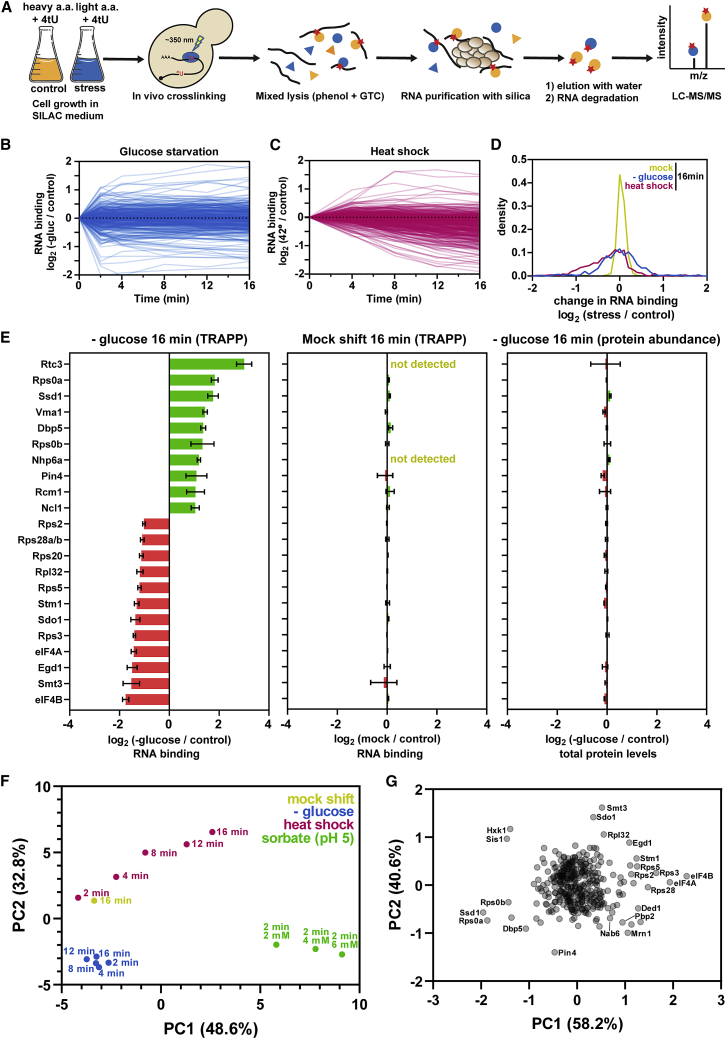
The Impact of Stress on the Yeast RNA-Binding Proteome (A) Summary of the TRAPP protocol. See main text for details. 4tU, 4-thiouracil; GTC, guanidinium thiocyanate. (B) Time course showing changes in RNA association during glucose starvation for individual RBPs in TRAPP analyses. (C) Same as (B) but for heat shock. (D) Density plot showing changes in RNA binding at 16 min following a mock shift, glucose starvation, or heat shock. (E) Bar chart showing all proteins with greater than 2-fold change in RNA association after 16 min of glucose starvation (left) or a mock shift (center). The right-hand panel shows changes in protein abundance following 16 min of glucose starvation (right). Error bars represent standard deviation. (F) Principal-component analysis (PCA) showing differences between conditions and time points. Axis titles show the extent of variation explained by a given principal component. (G) PCA comparing the changes in RNA binding for individual proteins following heat shock, glucose starvation, or mock shift of 16 min. See also Figure S1.

We previously developed TRAPP as a method to characterize the global RNA-binding proteome (Shchepachev et al., 2019). Here, we applied TRAPP to assess RBP dynamics in response to glucose starvation or heat shock. An overview of the approach is shown in Figure 1A. Yeast cells were grown in the presence of the photoreactive nucleobase 4-thiouracil (4tU), which is incorporated into nascent RNA during transcription. Following labeling, the cultures were rapidly filtered and shifted to medium containing the nonfermentable carbon sources of glycerol and ethanol (glucose withdrawal) or to standard glucose medium pre-warmed to 42°C (heat shock). At defined time points after transfer (2, 4, 8, 12, and 16 min), cells were irradiated with 350 nm UV light to induce crosslinks between 4tU-labeled RNAs and interacting RBPs. For comparison, cells were also irradiated prior to transfer (control) or transferred and irradiated without being subjected to stress (mock treated).

To quantify protein association with RNA, the TRAPP protocol also incorporates stable isotope labeling in cell culture (SILAC). Control cells were grown in media containing ^13^C_6_ (“heavy”) arginine and lysine, although stressed cells were cultured with standard amino acids (“light”). Stressed and control cultures were combined in equal proportion following irradiation and lysed together under denaturing conditions. Subsequently, the cleared cell lysate was incubated with silica beads, which bind RNA along with any crosslinked protein. Following elution of the RNA:protein complexes, the RNA component was degraded and the remaining protein was analyzed by mass spectrometry.

After filtering for proteins previously identified as high-confidence RNA binders (Shchepachev et al., 2019), we quantified the association of 338 proteins during glucose starvation and 399 following heat shock across all time points (Figures 1B and 1C). Most RBPs showed similar RNA association before and after glucose withdrawal; in total, only 22 proteins showed a greater than 2-fold shift in RNA binding by 16 min (Figure 1E; Table S2). Heat shock induced a more extensive response, with a greater bias toward loss of RNA binding (Figures 1C, 1D, and S1A). In contrast to the two stresses, a mock shift for 16 min produced no substantial changes in RNA binding (Figures 1D, 1E, and S1A). As an additional control, we also measured total protein levels before and after each stress (Figures 1E, S1A, and S1B; Table S3). RBP abundance was generally constant, suggesting that the observed differences in TRAPP recovery are attributable to changes in RNA association.

In general, glucose starvation caused more rapid changes in the RNA-protein interactome compared to heat shock. Many RBPs changed dramatically within the first 2 min following glucose depletion and were unchanged thereafter (Figures 1B and S1D). By contrast, heat shock induced more gradual, progressive changes in RNA binding throughout the time course (Figure 1C). These observations were further supported by principal-component analysis (PCA) (Figure 1F). All of the glucose starvation time points clustered close together, indicating a high degree of similarity following the initial rapid response. With heat shock, individual data points were more distinct in the PCA, showing a clear progression throughout the time course.

A decrease in intracellular pH has been suggested to underlie the response of yeast cells to multiple stresses (Dechant et al., 2014; García et al., 2017; Munder et al., 2016; Triandafillou et al., 2020). We therefore tested the effects of sorbic acid, a protonophore that equilibrates the cytosolic and extracellular pH. Cell cultures were incubated for 2 min in medium buffered at pH 5, together with increasing concentrations of sorbic acid (2, 4, and 6 mM) that are expected to correlate with decreasing intracellular pH (Munder et al., 2016). We observed substantial remodeling of the RBPome following sorbic acid treatment, including reduced binding of eIF4A and eIF4B (see below; Figure S1C). Overall, however, the changes seen following sorbate treatment were distinct from either glucose starvation or heat shock (Figures 1F and S1C; Table S2). We conclude that pH-mediated signaling is not the major contributor to the changes in RNA binding observed during these stresses. However, we cannot exclude the possibility that sorbate treatment induces stress effects in addition to the change in pH.

PCA analyses were also used to assess changes across the proteome for the glucose withdrawal and heat shock data (Figure 1G). A relatively small number of proteins were clearly outliers in their response to stress. Specific RBPs will be discussed below, but we note that a group of proteins showing strongly altered RNA binding, particularly Pin4, Mrn1, Pbp2, and Nab6, remain relatively uncharacterized (Hogan et al., 2008; Riordan et al., 2011). Their identification shows the potential value of TRAPP in providing initial functional data on uncharacterized factors. Further analyses of these proteins will be reported elsewhere.

### Glucose Starvation and Heat Shock Have Distinct Effects on Ribosome Biogenesis

**Figure 2:**
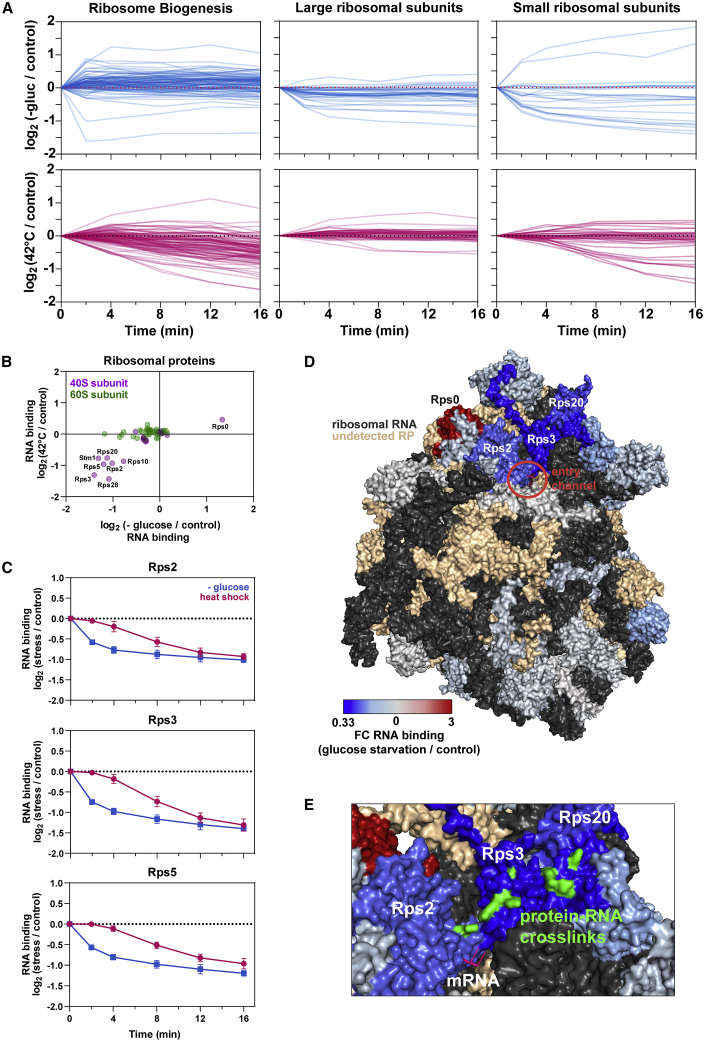
Changes in RNA Binding among Ribosomal Proteins (A) Time course showing changes in RNA binding during glucose withdrawal (upper; blue lines) and heat shock (lower; red lines) for various classes of RBPs in TRAPP analyses. Included in the figure are ribosome biogenesis factors (left), large ribosomal subunits (center), and small ribosomal subunits (right). (B) Scatterplot comparing the effects of glucose starvation and heat shock at 16 min on RNA binding for ribosomal proteins. (C) Time course showing RNA association for Rps2, Rps3, and Rps5. (D) Crystal structure (PDB: 3J77) of the yeast ribosome highlighting the changes in RNA association for each detected ribosomal protein. (E) A closeup view of the mRNA entry channel with amino acid-RNA crosslinking sites highlighted in green.

Strikingly, most ribosome maturation factors showed no appreciable drop in RNA binding in response to glucose starvation (Figure 2A), suggesting that glucose withdrawal rapidly pauses ribosome maturation and stabilizes the pre-rRNA. A substantial drop in RNA binding was seen only for Sdo1 (Figure S1D), which catalyzes the final step of 60S maturation (Kargas et al., 2019). Heat shock, by contrast, induced a progressive decrease in RNA binding over the 16-min time course for nearly all maturation factors. This indicates that the inhibition of ribosome synthesis develops over time, with maturation or degradation of the nascent particles. Notably, Sdo1 was again a rare exception, showing modest but significantly increased RNA association, potentially reflecting accumulation of very late pre-ribosomes immediately prior to final maturation of 60S subunits.

### Ribosomal Protein Binding Dynamics in Response to Cell Stress

A number of ribosomal proteins (RPs) showed decreased RNA association following either glucose withdrawal or heat shock (Figure 2A). The same set of RPs from the 40S subunit were altered in each stress (Figure 2B), but glucose starvation induced more rapid responses (for examples, see Figure 2C). We mapped these TRAPP results onto the structure of the ribosome, with each protein colored according to its change in RNA binding at 16 min following glucose withdrawal (Figure 2D). Intriguingly, RPs with altered binding predominately clustered around the mRNA channel. Indeed, the subset of proteins that directly contact the translating mRNA (e.g., Rps3) showed the most substantial drop in RNA association. We conclude that decreased translation during stress drives reduced RNA association for RPs that would otherwise contact the mRNA.

Finally, we mapped precise amino acid sites of RNA crosslinking to RPs, using published data from the iTRAPP method (Shchepachev et al., 2019). Visualization of these sites on the ribosomal structure (Figure 2E) revealed a succession of crosslinked amino acids along the surfaces of Rps2 (uS5) and Rps3 (uS3), apparently tracing the path of the mRNA as it approaches the channel. This is consistent with cryoelectron microscopy (cryo-EM) models of the yeast initiation complex (Llácer et al., 2018) and highlights the utility of TRAPP for capturing precise, *in vivo* interactions.

### A Common Set of Translation Initiation Factors Are Regulated in Response to Stress

We first confirmed that our strains and conditions recapitulate the reported translation inhibition following glucose withdrawal by polysome gradient analysis (Figure S2A; Ashe et al., 2000). Polysome levels were also reduced following heat shock but to a lesser extent (Figure S2A).

**Figure 3:**
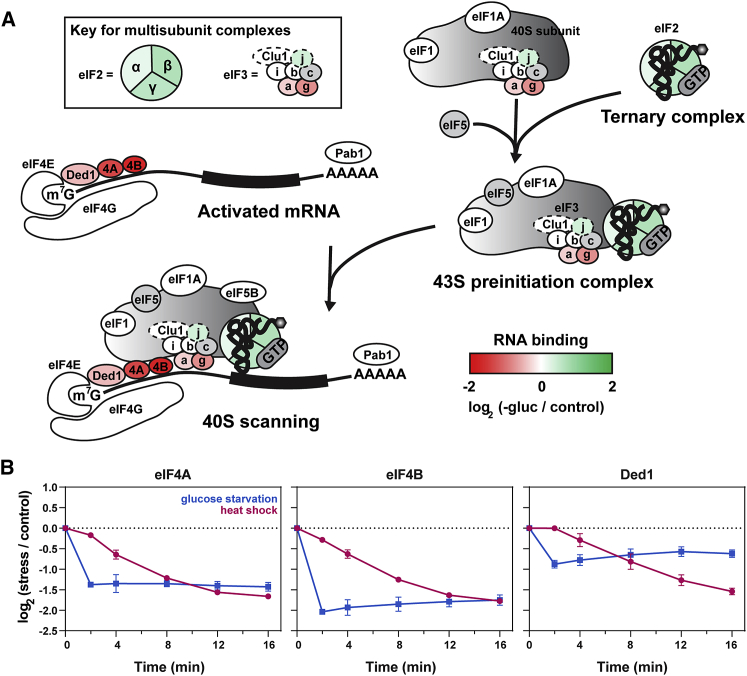
Changes in RNA Binding among Translation Initiation Factors (A) Overview of the translation initiation process. Each protein is colored according to its change in RNA association during glucose starvation in TRAPP analyses. Translation initiation factors shown in gray were not detected as RNA binding. (B) Time course showing changes in RNA binding for eIF4A, eIF4B, and Ded1 following glucose starvation (blue) and heat shock (pink) in TRAPP analyses. See also Figure S2B.

We next asked whether the TRAPP data could shed light on the mechanism of translational repression. We focused first on translation initiation, as it is the most common target of regulation. The RNA binding dynamics for each eIF are shown in Figure S2B. An overview of the translation initiation pathway is shown in Figure 3A (for details, see Introduction). Each protein is colored according to its change in RNA binding, specifically in response to glucose withdrawal, but most eIFs showed a consistent response to both stress conditions (Figure S2B).

We first considered the set of initiation factors primarily associated with the 40S subunit, including eIF1, eIF1A, eIF5, and eIF5B. These proteins remained associated with RNA following stress (Figures 3A and S2B), suggesting that their binding to the 40S subunit was unaltered. Next, we considered the eIF3 complex, subunits of which predominately bind either the mRNA or 40S ribosomes. The eIF3g and eIF3a subunits predominately contact the mRNA during translation (Valášek et al., 2017) and showed decreased RNA association. Conversely, eIF3b and eIF3i, which only interact with the 40S ribosome, were unchanged. Clu1 was also unchanged in RNA binding, but its place in the complex, if any, is unclear (Vornlocher et al., 1999). Only eiF3j showed increased RNA association following both stresses. However, eIF3j is not a constitutive subunit of the yeast eIF3 complex and may primarily function in translation termination (Beznosková et al., 2013; Young and Guydosh, 2019).

These findings suggested that the 43S preinitiation complex assembles normally during stress. However, a downstream block in translation initiation may impair its recruitment to mRNA. We therefore assessed mRNA-specific initiation factors that recruit the 43S complex. The cap binding protein eIF4E was largely unaffected by either stress (Figure S2B), whereas RNA binding by eIF4G was unaltered by glucose withdrawal but modestly reduced with heat shock. More dramatic effects on RNA binding were seen for the scanning factors eIF4A and eIF4B (Figure 3B). RNA association was strongly decreased following either stress but with much faster kinetics during glucose withdrawal. These observations are consistent with the reported loss of eIF4A from polysomes following glucose withdrawal (Castelli et al., 2011). eIF4A is a “DEAD-box” ATP-dependent RNA helicase, in which ATPase activity is coupled to mRNA binding and unwinding. Notably, a related DEAD-box translation initiation factor, Ded1, also showed a robust decrease in RNA binding during both stresses, though this was significantly less pronounced for glucose withdrawal (see below).

When bound to mRNA, eIF4A, eIF4B, and Ded1 recruit the 43S preinitiation complex, and Ded1 additionally assists in scanning and start codon recognition (Andreou et al., 2017; Guenther et al., 2018; Sen et al., 2015; Walker et al., 2013). Loss of RNA binding by these factors is therefore expected to strongly impair translation initiation. We conclude that a specific block in PIC recruitment and/or mRNA scanning underlies the translation repression seen following either glucose withdrawal or heat shock.

### Differential RNA Binding by eIF4A, eIF4B, and Ded1 upon Stress

To further investigate the role of eIF4A, eIF4B, and Ded1 in translation shutoff, we mapped the RNA binding sites for each protein using CRAC. Strains were constructed in which eIF4A and Ded1 were expressed as N-terminal, FH-tagged (FLAG-Ala_4_-His_8_) fusion proteins and eIF4B was expressed with a C-terminal HF tag (His_8_-Ala_4_-FLAG), under control of the endogenous promoters. The fusion proteins each supported wild-type growth, indicating that they are functional. Actively growing cells expressing the fusion proteins were UV-irradiated at 254 nm for ∼4–6 s in a VariX crosslinker to covalently fix direct protein:RNA contacts. After stringent, tandem-affinity purification, partial RNase digestion, and radiolabeling, protein:RNA complexes were isolated using SDS-PAGE (Figure S3A). Subsequently, crosslinked RNA fragments were amplified using RT-PCR and analyzed by high-throughput sequencing. For each protein, we collected datasets from unstressed cells (“control”), mock-shifted cells without stress (16 min), and following glucose withdrawal (30 s and 16 min) or heat shock (16 min; Figure S3B; Tables S4, S5, and S6). Metaplots of individual replicates showed good reproducibility (Figure S3D), and for subsequent analyses, replicate datasets were merged to provide improved coverage along individual transcripts.

We first examined interactions between each translation factor and ribosomal RNA in control cells (Figures S4A and S4B). eIF4A showed weak binding throughout 18S, together with two sharp peaks in 25S. However, both crosslinking sites were buried within the ribosome so likely represent sequencing artifacts. Clear results were seen with eIF4B, for which we observed a single major crosslinking site, situated close to the mRNA exit channel (nucleotide 1,060). Ded1 also crosslinked mainly to 18S rRNA, with prominent peaks near the mRNA entry (nucleotide 492) and exit (nucleotide 1,053) channel and additional binding at position 719. Crosslinking at all three sites is consistent with previously reported interactions between Ded1 and 18S rRNA (Guenther et al., 2018).

**Figure 4:**
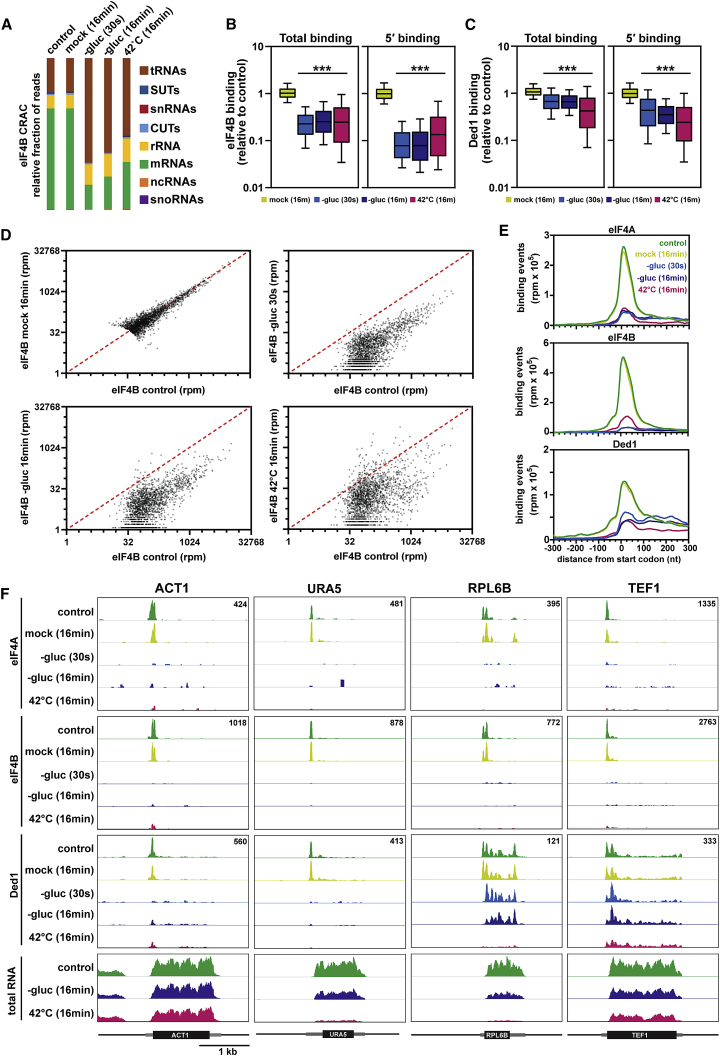
Genome-wide Analysis of the RNA Binding Profiles of eIF4A, eIF4B, and Ded1 (A) Breakdown of eIF4B-bound RNAs by biotype in CRAC analyses. (B) Boxplot showing the changes in eIF4B binding to individual mRNAs following either mock shift or stress in CRAC analyses. Each box represents the median with 25^th^ and 75^th^ percentiles. The whiskers show the 10^th^ and 90^th^ percentiles. ^∗∗∗^p < 10^−15^ relative to mock, using the unpaired t test to compare means. For (B)–(E), all analyses are based on a set of 2,000 transcripts that show the strongest binding to eIF4B in unstressed conditions. (C) Same as (B) but for Ded1. (D) Scatterplots comparing the changes in RNA binding between control and either mock shift (16 min), glucose starvation (30 s), glucose starvation (16 min), or heat shock (16 min). (E) Metaplots showing the distribution of eIF4A, eIF4B, and Ded1 binding around the mRNA start codon in CRAC analyses. (F) Binding of eIF4A, eIF4B, and Ded1 across the *ACT1*, *URA5*, *RPL6B*, and *TEF1* mRNAs. Each set of tracks is normalized to total library size using reads per million, with the exact value indicated in the upper right corner of each box. RNA-seq traces are shown at the bottom as a control. Each track is normalized to a spike-in control and thus represents the absolute abundance of each mRNA compared to the control. Each box represents a 3-kb window; a scale bar is shown at the bottom. The open reading frames (ORFs) are indicated as black boxes, with UTRs as flanking gray boxes of intermediate thickness. See also Figures S3–S5.

A breakdown of crosslinked RNAs by biotype revealed substantial differences pre- and post-stress. In unstressed cells, eIF4B primarily targeted mRNAs, but this enrichment was abruptly lost following exposure to either stress (Figure 4A). By contrast, cells subjected to a mock shift were indistinguishable from the control, indicating that the experimental protocol per se did not significantly perturb the cells. Similar but less dramatic trends were seen for Ded1 and eIF4A (Figure S3C). Analysis of binding sites on individual mRNAs provided a high-resolution snapshot of translation dynamics. Figures 4B–4D analyze 2,000 transcripts showing the strongest binding to eIF4B in unstressed conditions. The vast majority of transcripts showed decreased binding by eIF4B, Ded1, and eIF4A upon exposure to stress. Remarkably, binding was greatly decreased within the first 30 s of glucose withdrawal, consistent with prior reports that translation initiation is repressed within the first minute (Ashe et al., 2000). Binding was reduced across mRNAs, but the reduction was substantially more pronounced when considering only 5′ binding, defined as reads mapping to either the 5′ UTR or the first 150 nt of the open reading frame (Figures 4B–4D). This specific loss of 5′ binding was also seen in a metagene analysis of the top 2,000 bound mRNAs (Figures 4E and S3D). Heatmaps of the distribution of eIF4B along each of the 2,000 mRNAs confirmed that loss of 5′ binding is a general feature (Figure S3E; Table S7).

Having examined the “average” binding profile by metaplot, we next investigated how binding varied between individual mRNAs (Figures 4F and S5A). On most mRNAs, eIF4A and eIF4B targeted a single site close to the start codon (shown for *TEF1* and *URA5*). For a minority of transcripts, we observed two distinct peaks, usually on either side of the start codon (shown for *ACT1* and *RPL6B*). Overall, the two translation factors displayed a strikingly similar binding profile, consistent with eIF4B’s role as a cofactor for eIF4A (Andreou et al., 2017). In agreement with the metagene analysis, most individual transcripts showed sharply reduced binding to both eIF4A and eIF4B following stress.

Ded1 displayed a more complicated binding pattern. For most transcripts, including *ACT1* and *URA5* (Figure 4F), Ded1 showed a similar distribution to eIF4A and eIF4B, binding at a single site near the 5′ end in unstressed cells and largely dissociating during stress. However, a substantial fraction of transcripts showed pervasive binding throughout the length of the mRNA. To quantify this difference, we generated heatmaps in which individual transcripts were sorted by their ratio of 5′ versus pervasive binding. For Ded1, 66% of transcripts showed at least as much binding at downstream sites as at the 5′ end (Figures S5B and S5D). By contrast, only 5% of transcripts showed a comparable ratio for eIF4B (Figures S5C and S5D). The *RPL6B* mRNA illustrates these points well (Figure 4F). In unstressed cells, Ded1 was bound throughout the transcript, with modest enrichment at the 5′ end. Intriguingly, 5′ binding was selectively lost in response to glucose withdrawal, although downstream binding was maintained. By contrast, heat shock resulted in a general loss of Ded1 binding across the length of the mRNA. Similar results were seen for other transcripts, including *TEF1* (Figure 4F) and *RPL34B* (Figure S5A), albeit to varying degrees. At the metagene level, we observed a specific reduction in 5′ binding, although downstream binding was relatively unaltered following glucose withdrawal but decreased following heat shock (Figure 4E). Importantly, these observations are consistent with the TRAPP data, which showed that glucose withdrawal had a relatively modest effect on binding of Ded1 to RNA in comparison to heat shock (Figure 3B).

For most mRNA species, targeting by Ded1 was approximately proportional to transcript abundance, but there were a number of outliers (Figure S5E). The most prominent was the *DED1* mRNA itself, which was highly bound by Ded1. Intriguingly, most binding was concentrated within the 3′ UTR (Figure S5A), a pattern counter to most other mRNAs and suggestive of some form of auto-regulatory control.

### Heat Shock Triggers General mRNA Decay

**Figure 5:**
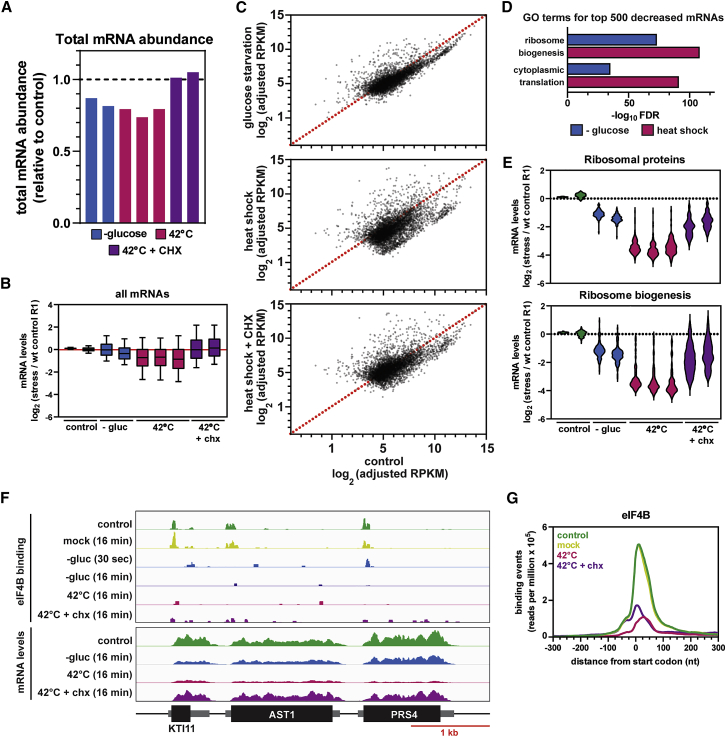
Global Analysis of mRNA Levels in Response to Stress (A) Bar graph showing the change in total mRNA abundance relative to an *S. pombe* spike-in control following glucose withdrawal (blue), heat shock (red), or heat shock plus cycloheximide (purple) for 16 min. (B) As in (A) but with boxplots showing changes in the abundance for 5,000 individual mRNAs. All samples were normalized to a single control sample (not shown). (C) Scatterplots comparing mRNA levels following glucose starvation (upper), heat shock (middle), or heat shock plus cycloheximide (lower) for 16 min relative to control. Points below the dotted red line indicate mRNAs with reduced abundance following stress. Each plot includes the 5,000 most-abundant mRNAs. RPKM (reads per kilobase per million) values were adjusted to account for the spike-in control. (D) GO (gene ontology) term enrichment among the 500 most-decreased mRNAs for each stress. (E) Violin plots showing the changes in mRNA levels for ribosomal protein (RP) mRNAs (upper) or ribosome biogenesis (RiBi) mRNAs (lower). (F) CRAC analysis showing binding of eIF4B across a selected genomic region (upper). RNA-seq tracks are normalized to a spike-in control and thus represent the absolute abundance of each mRNA compared to the control (lower). A scale bar is shown at the bottom. The ORFs are indicated as black boxes, with UTRs as flanking gray boxes of intermediate thickness. (G) Metaplots showing the distribution of eIF4B binding around the mRNA start codon. See also Figures S5 and S6B.

Our results indicate that specific translation initiation factors dissociate from mRNAs in response to stress. To control for changes in mRNA levels, we harvested RNA before and 16 min after each stress and performed RNA sequencing (RNA-seq), with RNA from *Schizosaccharomyces pombe* included as a spike-in control for quantitation. Replicates collected for each condition showed excellent reproducibility (Figure S6A; Table S8). Glucose withdrawal reduced overall mRNA abundance by 15%–20%, although heat shock reduced mRNA levels by 20%–25% (Figure 5A). The reduction in total mRNA was much less than the reduction in RNA binding by eIF4A, eIF4B, and Ded1 (Figure 3B), showing that the decreased initiation factor binding was not due to reduced mRNA abundance. This conclusion was further supported by analysis of individual mRNAs (Figure S6B).

Most mRNAs were only mildly affected by glucose depletion but were substantially decreased in response to heat shock (Figures 5B and 5C). GO analysis of the 500 most-depleted mRNAs revealed strong enrichment for translation-associated transcripts, primarily components of the ribosome biogenesis and cytoplasmic translation machinery (Figure 5D). This enrichment was seen for both stresses but was more significant following heat shock. We confirmed these results with quantitation of mRNAs that either encode ribosomal proteins or fall into the ribosome biogenesis (RiBi) regulon (Figure 5E), a group of over 200 coordinately regulated factors (Jorgensen et al., 2004; Klinge and Woolford, 2019; Wade et al., 2006). Both groups of mRNAs were, on average, reduced ∼2.5-fold by glucose depletion and ∼16-fold following heat shock (Figure 5E).

**Figure 6:**
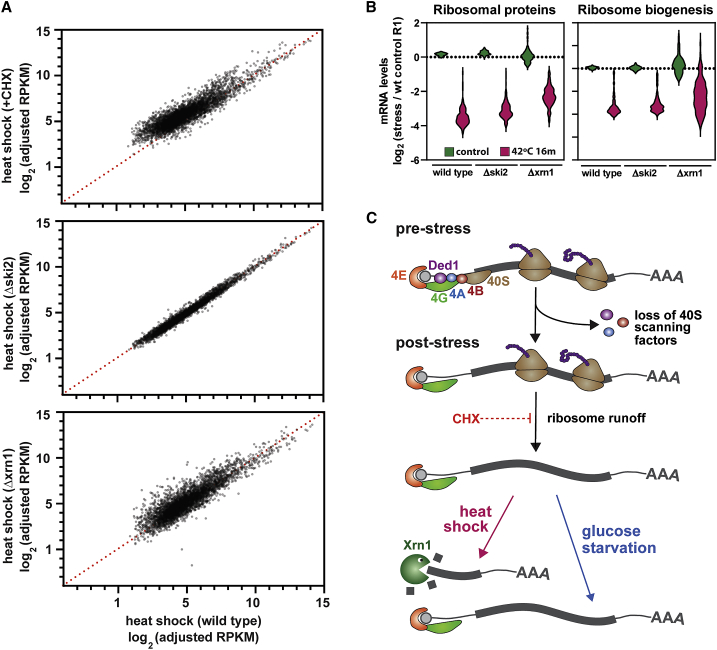
Cycloheximide Treatment or xrn1Δ Inhibits mRNA Decay during Heat Shock (A) Scatterplots comparing mRNA levels following heat shock for 16 min (x axis) relative to heat shock plus cycloheximide (upper); heat shock in a strain lacking Ski2 (middle); heat shock in a strain lacking Xrn1 (lower). Each plot includes the 5,000 most-abundant mRNAs. (B) Violin plots showing changes in mRNA levels among transcripts encoding ribosomal protein or ribosome biogenesis factors. (C) Model of the translational response to glucose starvation and heat shock. Upon exposure to either stress, the 40S scanning factors eIF4A, eIF4B, and Ded1 dissociate from the 5′ end of mRNAs, halting translation initiation. Already-initiated ribosomes continue translating before eventually terminating, potentially leaving “naked” mRNAs unprotected by the translational machinery. In the case of heat shock, Xrn1 is involved in degradation of a subset of these transcripts. With glucose starvation, by contrast, most mRNAs remain relatively stable.

The stability of a given mRNA species is often determined by competition between translation and degradation (Chan et al., 2018; Huch and Nissan, 2014; Schwartz and Parker, 1999). We therefore tested whether the decrease in abundance of specific transcripts could reflect mRNA decay induced by the translation shutdown. To determine the role of translation, heat shock was combined with cycloheximide treatment, which freezes ribosomes in place by inhibiting translation elongation. Notably, treatment with cycloheximide largely prevented the drop in mRNA levels following heat shock (Figures 5A, 5C, and 6A). We conclude that ribosome-bound mRNAs are protected from rapid mRNA decay induced during heat shock. In the absence of cycloheximide, mRNA degradation may be triggered by the shutdown in translation initiation combined with the ensuing ribosome runoff, resulting in “naked” mRNAs susceptible to degradation factors.

This hypothesis predicts that translation initiation factors dissociate from mRNAs prior to degradation of the transcript. To test this idea, we examined eIF4B binding by CRAC following heat shock, either with or without cotreatment with cycloheximide (shown for individual mRNAs in Figure 5F and as metagene plot in Figure 5G). If translation factors dissociate prior to mRNA decay, then eIF4B should lose 5′ RNA binding during heat shock, even if mRNA levels are stabilized by cycloheximide. Indeed, eIF4B binding to specific mRNAs was sharply reduced following heat shock, even when the cells were cotreated with cycloheximide to maintain mRNA levels. Similar results were observed genome-wide (Figure 5G). We conclude that translation initiation shutoff occurs upstream of mRNA decay.

### Heat Shock Induces Preferential mRNA 5**′** Degradation

Finally, we investigated the factors required for heat-shock-induced mRNA decay. In the cytoplasm, mRNAs can be 5′ degraded by the 5′ → 3′ exonuclease Xrn1 and/or 3′ degraded by the 3′ → 5′ exonuclease activity of the exosome, which also requires the Ski2 helicase as a cofactor. Strains lacking either Xrn1 or Ski2 are viable, whereas loss of both activities induces synthetic lethality (Brown et al., 2000), reflecting redundancy in the degradation pathways. We separately deleted the genes encoding Xrn1 and Ski2 and tested whether the effects of heat shock were rescued in the mutant strains by RNA-seq, including *S. pombe* RNA as a spike-in control. Figure 6A shows the comparison of heat shock alone, with heat shock in combination with cycloheximide, loss of Ski2, or loss of Xrn1 for the top 5,000 mRNAs.

Cycloheximide treatment caused the expected increase in mRNA levels (Figure 6A). The absence of Xrn1 also increased the abundance of many mRNAs following heat shock, whereas loss of Ski2 had minimal effects. Analysis of the classes of mRNAs most affected by heat shock, RPs and RiBi factors, supported these conclusions (Figure 6B). Loss of Ski2 conferred only mild protection on RP mRNAs with little effect on RiBi mRNAs. Loss of Xrn1 stabilized RP mRNAs ∼2-fold, with stronger overall stabilization for RiBi mRNAs (Figure 6B). Heterogeneity between RiBi mRNAs, with some species showing reduced abundance, probably reflects the role of Xrn1 in normal pre-rRNA processing.

## Discussion

RNA-binding proteins (RBPs) are critically important in the cellular response to environmental stress. The TRAPP technique allows RBP dynamics to be followed quantitatively on short timescales and is thus well suited for monitoring global changes in the protein-RNA interactome during stress. Here, we used TRAPP to follow RBP dynamics in *S. cerevisiae* during glucose starvation and heat shock. We observed rapid and specific changes in RNA binding for dozens of proteins, with translation-associated factors among the most significantly altered. Taken together, our results shed light on the mechanism of translational repression in yeast.

Consistent with a general shutdown of translation in response to stress, ribosomal proteins surrounding the mRNA channel showed decreased RNA association (Figure 2). Similar findings have recently been reported for human cells exposed to arsenite stress (Trendel et al., 2019). Consistent with our observations, the human channel proteins hRPS3 and hRPS28 showed especially strong loss of RNA binding (Trendel et al., 2019). Arsenite triggered extensive ribosomal degradation, perhaps to further enforce the translation shutdown or as a means to remove damaged ribosomes. However, we found no evidence of ribosome degradation in response to stress in yeast. Indeed, the abundance of most proteins was completely unaffected by either stress (Figure S1), suggesting changes in RBP levels are not a significant driver of rapid translation shutoff in yeast.

Throughout eukaryotes, phosphorylation of the translation initiation factor eIF2α leads to translation shutdown in response to various stresses. However, both glucose starvation and heat shock trigger translational repression via different but largely undefined pathways (Ashe et al., 2000; Grousl et al., 2009). To characterize these pathways, we examined translation initiation factors for RNA binding dynamics. Initiation factors that specifically associate with the 40S subunit showed little change in RNA binding during either stress. In contrast, both stresses provoked a rapid loss of mRNA binding by factors involved in 43S complex recruitment and scanning (the RNA helicases Ded1 and eIF4A, as well as the eIF4A cofactor eIF4B). We propose that this prevents recruitment and/or scanning of the 43S PIC, blocking translation initiation (Figure 6C). RPs that interact with the mRNA during elongation (e.g., Rps2, 3, and 5) showed a slower reduction in RNA binding during glucose starvation (Figure 2), supporting the idea that already-initiated ribosomes can continue elongation (Ashe et al., 2000).

To understand the translation shutdown in more detail, we mapped the RNA binding sites for eIF4A, eIF4B, and Ded1 using CRAC. In unstressed cells, eIF4A and B primarily targeted the 5′ ends of mRNAs, consistent with their role in initiation. Recent work proposed Ded1 as a stress sensor, due to its ability to reversibly condense into phase-separated granules (Hondele et al., 2019; Iserman et al., 2020; Wallace et al., 2015). Granule formation was stimulated by heat shock or a drop in intracellular pH, suggesting a possible mechanism for translation inhibition (Iserman et al., 2020). Our CRAC data on Ded1 are consistent with this model, showing a general loss of interactions with mRNA translation initiation regions but retention of binding further 3′ along the transcript (Figures 4 and S5). The specific RNA targets for Ded1-mediated assembly into stress-induced granules remain unknown (Hilliker et al., 2011; Iserman et al., 2020), but transcripts with pervasive Ded1 binding are strong candidates.

Loss of eIF4A or eIF4B in yeast confers greater inhibition of general translation than inactivation of Ded1 (Sen et al., 2016). Like Ded1, eIF4B condenses into phase-separated granules during heat shock (Wallace et al., 2015), suggesting that both proteins function as independent stress sensors. In principal, the loss of eIF4B binding to mRNAs during stress could reflect the loss of either of the functionally interacting initiation factors, eIF4A or Ded1. However, the CRAC data are more consistent with eIF4A playing the major role in eIF4B recruitment.

Because heat shock and glucose withdrawal can each drive relocation of translation factors and mRNAs into phase-separated cytoplasmic granules, similar effects on RNA stability might have been anticipated. However, this was not the case. Following glucose withdrawal, mRNA levels were relatively stable, whereas heat shock induced a substantial decrease in abundance for many mRNAs. This could reflect transcriptional repression or reduced RNA stability (Arribere et al., 2011), but effects of transcription are expected to be minimal over short periods. Depletion was particularly strong for mRNAs encoding nucleolar ribosome synthesis factors or cytoplasmic ribosomal proteins. These mRNAs were reduced 10-fold or more in 16 min, demonstrating activated RNA degradation (Figure 5). The same mRNAs are transcriptionally repressed as part of the integrated stress response (Gasch et al., 2000), which presumably maintains prolonged repression of ribosome synthesis. Remarkably, most mRNAs were stabilized by cotreatment with the translation elongation inhibitor cycloheximide, indicating that ribosome-bound mRNAs are protected from degradation.

Taken together, our findings lead us to the model in Figure 6C: under normal circumstances (i.e., in the absence of cycloheximide), heat shock triggers a halt in cap-dependent translation initiation, followed by runoff of already-initiated ribosomes. Without the protection of polysomes, the resulting transcripts are subject to degradation by the exonuclease Xrn1 and perhaps additional pathways. Under non-stress conditions, cycloheximide traps mRNAs in polysomes, blocking relocation to P-bodies (Sheth and Parker, 2003, Teixeira et al., 2005), and stabilizes mRNAs through inhibition of decapping (Hilgers et al., 2006). We predict that this is also the case following heat shock.

This model is consistent with prior observations suggesting a competition between translation and mRNA decay. Mutations in translation initiation factors lead to increased deadenylation and decapping (Schwartz and Parker, 1999). Moreover, chemical inhibition of translation initiation results in rapid mRNA decay (Chan et al., 2018). We suggest that a similar phenomenon occurs in response to heat shock, with some transcripts subject to increased degradation in the absence of active translation. A major open question is how translation-related mRNAs are preferentially targeted during heat shock and why they remain relatively stable during the shift from glucose to an alternative carbon source (Figure 5). The translation shutoff in response to glucose withdrawal is, if anything, more severe than that seen with heat shock (Figure S2A). Cytoplasmic mRNA deadenylation is downregulated following glucose withdrawal, making it likely that glucose signaling pathways block degradation in addition to immediately blocking initiation. Identifying the key signaling factors and their downstream targets is now a priority.

### Limitations of Study

Current methods for characterizing the RNA-bound proteome, including TRAPP, do not distinguish RNA classes. So, for most proteins, it is unclear whether a reduced TRAPP signal represents a loss of binding to only a subset of targets, in the absence of follow-up CRAC analyses. A technique that allows the simultaneous identification of proteins and RNAs is a long-term goal for the field. In the CRAC analyses, the data are normalized based on reads per million. In consequence, only changes in relative, but not absolute, binding can be determined.

The mechanisms that drive translation factor release from mRNA 5′ regions during stress remain unclear (Figure 4). For glucose starvation, this occurred remarkably quickly. Binding was completely ablated within 30 s, the earliest time point we could test. An unresolved question is how information on the depletion of glucose from the medium is gathered and transmitted to drive such rapid changes in protein binding. The proteins involved are extremely abundant; eIF4A (Tif1 plus Tif2) is present at ∼150,000 copies per yeast cell, comparable to the ribosome, with eIF4B (Tif3) and Ded1 at ∼25,000 copies (Ho et al., 2018). Presumably, extensive signal amplification is required to effectively regulate such abundant target proteins. Various signaling proteins and mRNA decay factors have been implicated in translation shutoff (Ashe et al., 2000; Coller and Parker, 2005; Holmes et al., 2004; Vaidyanathan et al., 2014), but their connections with the translation initiation machinery remain unknown.

## STAR★Methods

### Key Resources Table

**Table:** 

REAGENT or RESOURCE	SOURCE	IDENTIFIER
**Chemicals, Peptides, and Recombinant Proteins**		

SwaI	NEB	Cat#R0604S
BclI-HF	NEB	Cat#R3160S
Gel Extraction Kit	QIAGEN	Cat#28704
dNTPs	Takara	Cat#RR002M
Klenow exo-	NEB	Cat#M0212L
Uracil	Sigma-Aldrich	Cat#U0750-100G
Lysine	Sigma-Aldrich	Cat#L5626-100G
Arginine	Sigma-Aldrich	Cat#A5131-100G
^13^C_6_ lysine	CK Isotopes	Cat#CLM-2247-H
^13^C_6_ arginine	CK Isotopes	Cat#CLM-2265-H
Synthetic Dropout (-arg -lys -trp -ura)	Formedium	Cat#DCS1339
4-thiouracil	Sigma-Aldrich	Cat#440736-1G
Silicon dioxide	Honeywell	Cat#S5631-500G
Phenol pH 8	Sigma-Aldrich	Cat#P4557-400ML
RNase A/T1	Invitrogen	Cat#AM2286
Trypsin	Pierce	Cat#90057
cOmplete EDTA-free protease inhibitors	Roche	Cat#11873580001
Synthetic Dropout (-trp)	Formedium	Cat#DCS0149
Cycloheximide	Sigma-Aldrich	Cat#C7698-5G
FLAG peptide	Sigma-Aldrich	Cat#F3290-4MG
RNace-It	Agilent	Cat#400720
TSAP Thermosensitive Alkaline Phosphatase	Promega	Cat#M9910
RNasIN Ribonuclease inhibitor	Promega	Cat#N2511
T4 RNA Ligase I	NEB	Cat#M0204L
T4 RNA Ligase II truncated K227Q	NEB	Cat#M0351L
T4 PNK	NEB	Cat#M0201L
Proteinase K	Roche	Cat#03115836001
SuperScript III	Invitrogen	Cat#18080-044
La Taq	Takara	Cat#RR002M
MetaPhor Agarose	Lonza	Cat#50180

**Critical Commercial Assays**		

Ni-NTA Superflow	QIAGEN	Cat#30410
MinElute Gel Extraction Kit	QIAGEN	Cat#28606
NuPAGE Sample Loading Buffer	Invitrogen	Cat#NP0007
NuPAGE 4-12% Bis-Tris Gel	Invitrogen	Cat#NP0321BOX
NuPAGE MOPS Running Buffer	Invitrogen	Cat#NP0001-02
Mini-PROTEAN TGX Gels	Bio-Rad	Cat#4561093
Hybond-N+ nitrocellulose membranes	GE Healthcare	Cat#RPN303B
Gel Extraction kit	QIAGEN	Cat#28704
Imperial Protein Stain	Thermo-Scientific	Cat#24615
NEBNext poly(A) mRNA Magnetic Isolation Module	NEB	Cat#E7490
NEBNext Ultra II Directional RNA library prep kit for Illumina	NEB	Cat#7760
Empore SPE C18 disks	Sigma-Aldrich	Cat#66883-U
Zirconia Beads	Thistle Scientific	Cat#11079105z
anti-Flag beads	Sigma-Aldrich	Cat#M8823-1ML
Protein Lo-Bind tubes	Eppendorf	Cat#022431102

**Deposited Data**		

Raw image files	Mendeley	https://data.mendeley.com/datasets/fwf94d8cf7/1
CRAC and RNAseq raw data	NCBI Gene Expression Omnibus	GSE148166
Mass Spectrometry raw data	ProteomeXchange (PRIDE)	PXD019141

**Experimental Models: Organisms/Strains**		

S. cerevisiae background strain FH-DED1 (MATa his3Δ1 leu2Δ0 met15Δ0 ura3Δ0 FH-DED1)	This paper.	ySB131
S. cerevisiae background strain Δski2 (MATa his3Δ1 leu2Δ0 met15Δ0 ura3Δ0 Δski2)	This paper.	ySB151
S. cerevisiae background strain BY4741 (MATa his3Δ1 leu2Δ0 met15Δ0 ura3Δ0)	Euroscarf collection	ySB001
S. cerevisiae SILAC strain (MATa his3Δ1 leu2Δ0 met15Δ0 ura3Δ0 lys9Δ arg4Δ)	Shchepachev et al., 2019	ySB076
S. cerevisiae background strain Δxrn1 (MATa his3Δ1 leu2Δ0 met15Δ0 ura3Δ0 Δxrn1)	Euroscarf deletion collection	ySB082
S. cerevisiae background strain FH-TIF1 (MATa his3Δ1 leu2Δ0 met15Δ0 ura3Δ0 FH-TIF1)	This paper.	ySB123
S. cerevisiae background strain TIF3-HF (MATa his3Δ1 leu2Δ0 met15Δ0 ura3Δ0 TIF3-HF)	This paper.	ySB124

**Oligonucleotides**		

	See Table S1	N/A

**Recombinant DNA**		

pML104-DED1 targeting gRNA	This paper.	pSB064
pML104-SKI2 targeting gRNA	This paper.	pSB077
pML107	Laughery et al., 2015	Cat#67639 (Addgene)
pML104-TIF3 targeting gRNA	This paper.	pSB051
pML107-TIF1 targeting gRNA	This paper.	pSB053
pML104	Laughery et al., 2015	Cat#67638 (Addgene)

**Software and Algorithms**		

Novoalign v2.07.00	Novocraft	http://www.novocraft.com/products/novoalign/
Integrated Genomics Viewer	Robinson et al., 2011	http://software.broadinstitute.org/software/igv/
Prism 8	GraphPad	https://www.graphpad.com/
pyCRAC	Webb et al., 2014	http://sandergranneman.bio.ed.ac.uk/pycrac-software
MaxQuant v1.6.1.0	Cox and Mann, 2008	https://www.maxquant.org/
Scikit-learn v0.22.2 library		https://scikit-learn.org/stable/
Bedtools v2.27.0	Quinlan and Hall, 2010	https://github.com/arq5x/bedtools2
Flexbar v3.4.0	Dodt et al., 2012	https://github.com/seqan/flexbar/releases/tag/v3.4.0
GOrilla	Eden et al., 2009	http://cbl-gorilla.cs.technion.ac.il/
BBduk	Department of Energy- Joint Genome Institute	https://sourceforge.net/projects/bbmap

### Resource Availability

#### Lead Contact

Further information and requests for resources and reagents should be directed to the Lead Contact, David Tollervey (d.tollervey@ed.ac.uk).

#### Materials Availability

Plasmids and strains generated in this study are available upon request.

#### Data and Code Availability

The accession number for all sequence data reported in this paper is GEO: GSE148166.

The proteomics data are available through the ProteomeXchange Consortium via the PRIDE (Perez-Riverol et al., 2019) partner repository with the dataset identifier PRIDE: PXD019141.

### Experimental Model and Subject Details

All *S. cerevisiae* strains used in this study were derived from the BY4741 background (MATa his3Δ1 leu2Δ0 met15Δ0 ura3Δ0). For SILAC experiments, we used a strain auxotrophic for lysine and arginine biosynthesis (BY4741 *Δlys9 Δarg4*) (Shchepachev et al., 2019).

### Method Details

#### Cell culture and medium

All yeast strains were cultured at 30°C in synthetic medium containing 2% glucose to 0.4 OD_600_. Cells were either harvested directly (control), or collected by filtration and transferred to medium containing glucose (mock shift), medium lacking glucose but containing 2% glycerol and 2% ethanol (glucose starvation), or to glucose-containing medium prewarmed to 42°C (heat shock). Cycloheximide was used at a concentration of 0.1 mg/mL.

#### Plasmid construction

All proteins were tagged using CRISPR-Cas9 as described (Laughery et al., 2015). eIF4B and Ded1 were tagged using the pML104 vector backbone (Addgene: 67638) while eIF4A was tagged using pML107 (Addgene: 67639). Both plasmids included an ampicillin resistance gene, a Cas9 expression construct, and a guide RNA (gRNA) cloning site, but differed in selectable marker (*URA3* and *LEU2*, respectively). Plasmid DNA was prepared from dam^**-**^ *E. coli.* For each plasmid, 10 μg were digested overnight with SwaI (NEB Cat#R0604S), and then for 2 h at 50°C with BclI-HF (NEB Cat#R3160S). The digested vector was purified by gel extraction (QIAGEN Cat#28704).

Guide RNA oligos were designed as reported (Laughery et al., 2015). Each oligo pair was annealed in a reaction consisting of 1 μM forward oligo, 1 μM reverse oligo, 50 mM Tris-HCl 7.5, 10 mM MgCl_2_, 1 mM ATP, and 10 mM DTT in a 100 μL reaction volume. The hybridization reaction was initially incubated at 95°C for 6 min, and gradually decreased to 25°C at the rate of 1.33°C/min. Hybridized substrates were then ligated into the digested vector at 25°C for 4 h. The ligation reaction consisted of 265 ng vector, 0.8 nmol insert, 50 mM Tris-HCl 7.5, 10 mM MgCl_2_, 1 mM ATP, 10 mM DTT, and 800 units of T4 DNA ligase (NEB M0202L) in a 40 μL reaction volume. The ligation mix was transformed into homemade DH5α *E. coli*, and plated overnight on LB-Amp. DNA was isolated from several colonies and sequenced to ensure correct insertion of the guide sequence.

#### Strain construction

For CRAC experiments, the chromosomal copies of *TIF1* (one of two genes encoding eIF4A) and *DED1* were N-terminally tagged with FH (Flag-His), consisting of a single Flag motif, a four-alanine spacer, and eight consecutive histidine residues (DYKDDDDKAAAAHHHHHHHH). *TIF3* (eIF4B) was C-terminally tagged with the same elements in reverse (HHHHHHHHAAAADYKDDDDK), the HF (His-Flag) tag. For the *DED1* tagging, we appended an additional amino acid (asparagine) to the N terminus of the tag to remove the gRNA cleavage site. All strains were generated using CRISPR as described below (Laughery et al., 2015).

To generate repair templates, we designed fragments consisting of the HF or FH DNA sequence flanked by 50 bp homology arms. Typically, synonymous mutations were used to disrupt the PAM site to prevent any further cleavage by Cas9. Each repair template was made by annealing two single stranded oligo nucleotides sharing 20 bp of complementarity at their 3′ ends. Each oligo pair was annealed in a reaction consisting of 10 μM forward oligo, 10 μM reverse oligo, 50 mM NaCl, 10 mM Tris-HCl 7.9, 10 mM MgCl_2_, and 100 μg/mL BSA in a 43 μL reaction volume. The hybridization reaction was initially incubated at 95°C for 6 min, and gradually decreased to 25°C at the rate of 1.33°C/min. Subsequently, the annealed oligos were incubated in the same buffer supplemented with 250 μM dNTPs (Takara Cat#RR002M) and 5U Klenow exo- (NEB Cat#M0212L) in a 50μL reaction at 37°C for 1 h to fill in the single stranded regions.

To tag the genes of interest, BY4741 yeast were transformed using the standard LiOAc protocol with 500 ng of gRNA plasmid and 10 pmol of the corresponding repair template. Transformants were plated onto either leu- or ura- medium. After three days, several clones from each transformation were plated again on selective medium, and allowed to grow for an additional 2-3 days. Single colonies were selected and plated on YPD for 2 days. Finally, individual colonies were grown overnight in liquid YPD and frozen. The clones were verified by PCR using flanking primers and confirmed by sequencing. The *SKI2* gene was deleted using a guide RNA targeting the end of the open reading frame using the same approach as with the tagging.

All yeast strains used in this study are listed in the Key Resources Table. DNA oligonucleotides and plasmids used for strain construction are listed in Table S1 and the Key Resources Table, respectively.

#### TRAPP

For TRAPP experiments, cells were cultured at 30°C in 700 mL of synthetic dropout (SD) -arg -lys -trp -ura (Formedium Cat#DCS1339), supplemented with 20 μg/mL uracil (Sigma-Aldrich Cat#U0750-100G) and 2% glucose. Light media additionally included 30 μg/mL lysine (Sigma-Aldrich L5626-100G), and 5 μg/mL arginine (Sigma-Aldrich Cat#A5131-100G), while heavy media included 30 μg/mL ^13^C_6_ lysine (CK Isotopes Cat#CLM-2247-H), and 5 μg/mL ^13^C_6_ arginine (CK Isotopes Cat#CLM-2265-H). For most experiments, light labeling was used for cells exposed to stress, while heavy labeling was used for control cells. Prior to each experiment, cells were cultured with heavy isotope for at least 8 generations to ensure complete labeling.

Overnight starter cultures were inoculated into fresh media at a starting OD_600_ of 0.05. At OD_600_ 0.15, 4-thiouracil (4tU) (Sigma-Aldrich Cat#440736-1G) was added to the media at a final concentration of 0.5 mM, and the cells were grown for another 200 minutes (approx. OD_600_ 0.4). After 4tU treatment for three hours, heavy labeled cells were collected by filtration and transferred to 700 mL of heavy media lacking 4tU. The UV lamps were allowed to warm up for one minute, before the shutters were opened and the cells were crosslinked for 38 s (350 nm; 7.3 J cm^−2^). Cells were harvested by filtration, resuspended in 50 mL ice-cold PBS, and 200 ODs were collected by centrifugation. The cell pellets were frozen for later processing.

Light-labeled cells were collected by filtration and quickly (3-4 s) transferred to medium containing 2% each of glycerol and ethanol instead of glucose (glucose starvation), or standard medium pre-warmed to 42°C (heat shock). Cells for each time point were grown separately and harvesting times were sufficiently staggered to allow enough time to collect each time point. For the earliest time point, the cells were transferred to the appropriate medium lacking 4tU and crosslinked at 2 min. The cells were then processed as described above for the control sample. For all other time points, the cells were first transferred to medium containing 4tU and cultured for the appropriate amount of time. Two min prior to the end point, the cells were collected by filtration and transferred to media lacking 4tU. The cells were then crosslinked and processed as described above.

In preparation for TRAPP, 10 g of silica sand (Honeywell Cat#S5631-500G) was left overnight in 50 mL of 1M HCl. The sand was then washed with 50 mL water three times (2,000 g; 2 min). After the final wash, the sand was resuspended in equivolume water to achieve a 50% slurry suspension.

Matching SILAC pairs were each resuspended in 1 mL of a 1:1 mix between phenol pH 8 (Sigma-Aldrich Cat#P4557-400ML) and GTC lysis buffer (4 M guanidine thiocyanate, 50 mM Tris-HCl pH 8.0, 10 mM EDTA, and 1% β-mercaptoethanol), and combined in equal proportion (400 ODs total in 2 mL phenol-GTC) in a 50 mL conical. Three mL of zirconia beads (Thistle Scientific Cat#11079105z) was added and the cells were vortexed for 6 min to lyse the cells. An additional 8 mL of phenol-GTC was added, and the cells were vortexed for an additional 1 min. The cell lysate was centrifuged at 4,600rpm in a Sorvall centrifuge for 5 min. The supernatant was transferred to several 2 mL Eppendorf tubes, and centrifuged at 16000 g for 10 min. The cleared lysate was pooled in a fresh 50 mL conical, and added to 0.1 volumes of 3 M sodium acetate pH 4.0 and mixed. An equal volume of ethanol was slowly added to the mix, followed by 1 mL of 50% silica slurry and an additional 500 μL of ethanol. The lysate was incubated at room temperature on a rotating wheel for 30 min.

The silica sand was washed three times at 2,000 rpm for 2 min with 10 mL of wash buffer I (4 M guanidine thiocyanate, 1 M sodium acetate pH 4, and 30% ethanol), followed by three washes with 10 mL of wash buffer II (100 mM NaCl, 50 mM Tris-HCl pH 6.4, and 80% ethanol). The silica was resuspended in ∼3.5 mL wash buffer II, transferred to two 2 mL Eppendorf tubes, and centrifuged at 2,000 g for 2 min. The supernatant was removed and the tubes were centrifuged in a SpeedVac for 20 min at 45°C to remove residual wash buffer.

Protein:RNA complexes were eluted three times with 10 mM Tris pH 8.0. For each elution, the silica was thoroughly resuspended in 500 μL of elution buffer, and incubated with shaking at 37°C for 5 min. The resulting eluates were combined and centrifuged at 20000 g to remove residual silica. The supernatant was removed, centrifuged a second time, and the resulting supernatant was transferred to protein LoBind tubes (Eppendorf Cat#022431102). The eluates were incubated with 0.25 μL of RNaseA/T1 (Invitrogen Cat#AM2286) for two hours at 37°C, followed by centrifugation overnight in a SpeedVac at room temperature.

The protein in each tube was resuspended in 35 μL of 1.5X Laemmli buffer (90 mM Tris-HCl pH 6.8, 3% SDS, 15% glycerol, and 8% β-mercaptoethanol). Matching samples were combined into a single tube, and incubated for 5 min at 100°C. Approximately 25 μL was loaded onto a 4%–20% Miniprotean TGX gel (Bio-Rad Cat#4561093) and run in Tris-Glycine running buffer. Individual samples were typically split across two lanes to avoid overloading. Each gel was run at 50 V for 40 min (approximately 2 cm), and then placed in a 15 cm Petri dish and rinsed with distilled water for approximately 30 min. Subsequently, the gel was stained with Imperial Protein Stain (Thermo-Scientific Cat#24615) for 1 h, rinsed several times with water, and allowed to destain in water for 3 h to overnight.

Protein smears were cut into two sections consisting of high- and low-molecular weight proteins. Each gel fragment was diced into smaller pieces roughly 1 mm^3^ in size and collected in a 1.5 mL Eppendorf tube. The gel pieces were destained in a solution consisting of 50 mM ammonium biocarbonate and 50% acetonitrile for 30 min at 37°C with shaking at 750 rpm.

Proteins were then digested with trypsin as described by Shevchenko et al. (1996). Briefly, proteins were reduced with 10 mM dithiothreitol in ammonium bicarbonate for 30 min at 37°C and alkylated with 55 mM iodoacetamide in ammonium bicarbonate for 20 min at ambient temperature in the dark. They were then digested overnight at 37°C with 13 ng/μL trypsin (Pierce Cat#90057).

For this and subsequent steps, enough solution was added to cover the gel pieces completely. Subsequently, the gel fragments were treated with acetonitrile for 5 min to further shrink them. The acetonitrile solution was removed, and disulfide bonds were reduced with 10 mM dithiothreitol in a 50 mM ammonium bicarbonate solution for 30 min at 37°C with shaking. The DTT was removed, and the gel pieces were again shrunk by 5 min incubation with acetonitrile. Subsequently, the gel fragments were treated with 55 mM iodacetamide and 50 mM ammonium bicarbonate to alkylate free cysteines. The samples were digested overnight with trypsin in buffer consisting of 10 mM ammonium bicarbonate and 10% acetonitrile.

Following trypsin digestion, the samples were acidified to pH 1-2 using 10% trifluoroacetic acid (TFA) and processed using the stage-tip method (Rappsilber et al., 2007). Briefly, three C-18 discs (Sigma-Aldrich Cat#66883-U) were cut out and placed in a 200 μL pipet tip with gentle compression. Each stage tip was placed in a 1.5 mL collection tube with a hole in the lid to hold the stage tip. The stage tips were conditioned with washes of 40 μL methanol followed by 80 μL of 0.1% TFA. Subsequently, the peptide solution was loaded on the stage tip and centrifuged at 1000 g, to allow the peptide to bind the column. Once all of the solution had passed through, the stage tips were loaded with 25 μL of 0.1% TFA, and temporarily placed at 4°C.

In parallel, the remaining gel fragments were incubated for 10 min in a solution consisting of 80% ACN and 0.1% TFA in order to remove any remaining peptides from the gel. This solution was then transferred to a 2 mL Protein LoBind tube and dried under vacuum centrifugation at 60°C. Afterward, the protein pellet was resuspended in 200 μL of 0.1% TFA and passed through the stage tip. Finally, the stage tip was washed twice with 100 μL of 0.1% TFA, and then placed at −20°C for storage prior to mass spectrometry.

#### Mass Spectrometry

Following digestion, samples were diluted with equal volume of 0.1% Trifluoroacetic acid (TFA) and spun onto StageTips as described by . Peptides were eluted in 40 μL of 80% acetonitrile in 0.1% TFA and concentrated down to 5 μL by vacuum centrifugation (Concentrator 5301, Eppendorf, UK). The peptide sample was then prepared for LC-MS/MS analysis by diluting it to 5 μL by 0.1% TFA. MS-analyses were performed on an Orbitrap Fusion™ Lumos™ Tribrid™ mass spectrometer (Thermo Fisher Scientific, UK), coupled on-line, to Ultimate 3000 RSLCnano Systems (Dionex, Thermo Fisher Scientific). Peptides were separated on a 50 cm EASY-Spray column (Thermo Fisher Scientific, UK) assembled in an EASY-Spray source (Thermo Fisher Scientific, UK) and operated at a constant temperature of 50°C.

Mobile phase A consisted of water and 0.1% formic acid (Sigma Aldrich, UK); mobile phase B consisted of 80% acetonitrile and 0.1% formic acid. The total run time per fraction was 190 min and for protein abundance samples was 160 min per fraction. Peptides were loaded onto the column at a flow rate of 0.3 μL min^-1^ and eluted at a flow rate of 0.25 μL min^-1^ according to the following gradient: 2 to 40% buffer B in 150 min, then to 95% in 16 min. For protein abundance samples the gradient was 2 to 40% mobile phase B in 120 min and then to 05% in 16 min. In both cases, samples were subjected to mass spectrometry analysis under the same conditions. Specifically, survey scans were performed at resolution of 120,000 in the orbitrap with scan range 400-1,900 m/z and an ion target of 4.0e5. The RF lens was set to 30% and the maximum injection time to 50ms. The cycle time was set to 3 s and dynamic exclusion to 60 s. MS2 was performed in the Ion Trap at a rapid scan mode with ion target of 1.0E4 and HCD fragmentation with normalized collision energy of 27 (Olsen et al., 2007). The isolation window in the quadrupole was set at 1.4 Thomson and the maximum injection time was set to 35 ms. Only ions with charge between 2 and 7 were selected for MS2.

#### Total proteomics

Cell culturing was performed as described above for the TRAPP experiments. Approximately 50 ODs of cells were resuspended in 200 μL of TN150 (50 mM Tris-HCl pH 7.5, 150 mM NaCl, 0.1% NP-40, 5 mM β-mercaptoethanol and a cOmplete EDTA-free protease-inhibitor cocktail (Roche Cat#11873580001) (1 tablet / 50 mL) and added to 500 μL of zirconia beads in a 1.5 mL Eppendorf tube. The cells were lysed with five one-minute pulses, with cooling on ice for one minute in between. The lysate was further diluted with 0.6 mL TN150, briefly vortexed, and combined 1:1 with 3X Laemmli buffer. The sample was incubated at 100°C for five minutes and centrifuged at 20,000 g for 1 min. Approximately 10 μL of supernatant was loaded onto a 4%–20% Miniprotean TGX gel and run in Tris-Glycine running buffer at 100 V. Subsequently, the gel was washed with water, stained with Imperial Protein Stain for 1 h, rinsed several times with water, and allowed to destain in water overnight. Each lane was divided cut into six fractions, and then processed as described above for TRAPP.

#### RNAseq

*S. cerevisiae* BY4741 cells were grown to 0.4 OD in SD -trp (Formedium Cat#DCS0149) media. For control samples, the cells were collected by filtration, transferred to 50 mL ice-cold PBS, and centrifuged. Cell pellets were frozen for later use. For stress samples, cells were collected by filtration and transferred to the appropriate medium for 16 min, collected by filtration, and frozen at −80°C. Biological duplicates were collected for each condition. *Schizosaccharomyces pombe* 972H cells were harvested separately and frozen for use as a spike-in control. RNA from all samples was purified using phenol:chloroform extraction. *S. pombe* RNA was spiked into each sample at a final concentration of 2%. Libraries for RNAseq were prepared by the Wellcome Trust Clinical Research Facility at Western General Hospital (Edinburgh, UK) using the poly(A) mRNA magnetic isolation kit (NEB Cat#E7490) and the NEBNEXT Ultra II Directional RNA Library Prep kit (NEB Cat#7760). The libraries were sequenced using Next-Seq with single-end, 75nt output.

#### Polysome profiling

The technique used was modified from Winz et al. (2019). Overnight cultures of yeast were diluted to 0.05 in 100 mL of SC -trp medium and cultured with shaking at 30°C. At OD_600_ 0.4, the cells were treated with 0.1 mg/mL cycloheximide (Sigma-Aldrich Cat#C7698-5G) for 2 minutes. The cells were collected by filtration and resuspended in 50 mL of ice-cold PBS supplemented with cycloheximide. The cells were then pelleted at 4600 rpm for 2 min and stored at −80°C.

For lysis, cells were resuspended in 200 μL of buffer (20 mM HEPES-KOH, 7.4; 100 mM KOAc; and 2 mM MgOAc) and transferred to 2 mL tubes containing 200 μL of zirconia beads. Cells were lysed with five one-minute vortexing pulses, with cooling on ice in between rounds. The lysate was diluted with an additional 200 μL of lysis buffer, and the combined supernatant was transferred to a new Eppendorf tube. The lysate was cleared using two rounds of centrifugation at max speed for 5 min each.

Approximately 20 OD_260_ of cells were loaded onto 10%–45% sucrose gradients in 1X gradient buffer (10 mM Tris-acetate, pH 7.4, 70 mM ammonium acetate, 4 mM magnesium acetate) prepared using the Gradient Master (BioComp). Subsequently, the gradients were centrifuged in an SW40-Ti rotor in an Optima XPN-100 Ultracentrifuge (Beckman Coulter) at 38,000 rpm for 2.5 h at 4°C. Absorbance profiles were visualized using the Piston Gradient Fractionator (BioComp).

#### CRAC

The CRAC protocol is based on Granneman et al. (2011), with some modifications. Most importantly, we substituted the two Protein A affinity tags and the TEV cleavage site with a single Flag tag. The histidine tag was lengthened from six residues to eight.

For each CRAC experiment, 700 mL of cells were grown in SC -trp media. At OD_600_ 0.4, the cells were UV-irradiated at 254 nm with a dose of 100 mJ/cm^2^ (4-6 s) using the Vari-X-Link crosslinker (McKellar et al., 2020; van Nues et al., 2017). Following crosslinking, cells were collected by filtration and resuspended in 50 mL ice-cold PBS, and then centrifuged at 4,600 g for 2 min. The cell pellets were stored at −80°C.

Cell pellets were resuspended in 500 μL TN150 (50 mM Tris-HCl pH 7.5, 150 mM NaCl, 0.1% NP-40, 5 mM β-mercaptoethanol and a protease-inhibitor cocktail (1 tablet / 50 mL) and added to 1.25 mL of zirconia beads in a 50 mL conical. The cells were lysed with five one-minute pulses, with cooling on ice in between. The lysate was further diluted with 1.5 mL TN150, briefly vortexed, and centrifuged at 4,600 g for 5 min. The supernatant was transferred to Eppendorf tubes and spun for an additional 20 min at 16,000 g. In parallel, 100 μL of magnetic anti-Flag bead slurry (Sigma-Aldrich Cat#M8823-1ML) was washed twice with TN150. The cleared lysate was incubated with the anti-Flag beads for two hours at 4°C, with nutating. Subsequently, the beads were washed four times with TN150 (5 min nutating at 4°C) and then incubated with 200 μL of flag peptide (Sigma-Aldrich Cat#F3290-4MG) (100 μg / mL in TBS) at 37°C with shaking for 15 min. The eluate was transferred to a fresh tube containing 350 μL TN150 and treated with RNace-IT (Agilent Cat#400720) (0.1U, 5 min, 37°C) to fragment protein-bound RNA. The RNase reaction was quenched by transferring the eluate to a tube containing 400 mg guanidine hydrochloride. The solution was adjusted for nickel affinity purification with the addition of 27 μL NaCl (5 M) and 3 μL imidazole (2.5 M) and added to 50 μL of washed nickel beads (QIAGEN Cat#30410).

Following an overnight incubation, the nickel beads were transferred to a spin column and washed three times with 400 μL WBI (6.0 M guanidine hydrochloride, 50 mM Tris-HCl pH 7.5, 300 mM NaCl, 0.1% NP-40, 10 mM imidazole, and 5 mM β-mercaptoethanol), and then three times with 600 μL 1xPNK buffer (50mM Tris-HCl pH 7.5, 10 mM MgCl_2_, 0.5% NP-40, and 5 mM β-mercaptoethanol). Subsequent reactions (80 μL total volume for each) were performed in the columns, and afterward washed once with WBI and three times with 1xPNK buffer:

1. Phosphatase treatment (1x PNK buffer, 8 U TSAP (Promega, Cat#M9910), 80 U RNasIN (Promega Cat#N2511); 37°C for 30 min).
2. 3′ linker ligation (1x PNK buffer, 20 U T4 RNA ligase I (NEB Cat#M0204L), 20 U T4 RNA Ligase II truncated K227Q (NEB Cat#M0351L), 80 U RNasIN, 1 μM preadenylated 3′ miRCat-33 linker (IDT); 25°C for 6 h).
3. 5′ end phosphorylation and radiolabeling (1x PNK buffer, 40 U T4 PNK (NEB Cat#M0201L), 40 μCi ^32^P-γATP; 37°C for 60 min, with addition of 100 nmol of ATP after 40 min).
4. 5′ linker ligation (1x PNK buffer, 40 U T4 RNA ligase I, 80 U RNasIN, 5′ linker, 1 mM ATP; 16°C overnight).

The beads were washed twice with WBII (50 mM Tris-HCl pH 7.5, 50 mM NaCl, 0.1% NP-40, 10 mM imidazole, and 5 mM β-mercaptoethanol). Protein:RNA complexes were eluted twice (10 minutes each) in 40 μL of elution buffer (same as WBII but with 300 mM imidazole). At this point, different replicates or conditions for the same protein were combined. The merged eluates were precipitated with 5X volume acetone at −20°C for at least two hours. RNPs were pelleted at 16000 g for 20 min, and resuspended in 20 μL 1X NuPAGE sample loading buffer (Invitrogen Cat#NP0007) supplemented with 8% β-mercaptoethanol. The sample was denatured by incubation at 65°C for 10 min, and run on a 4%–12% Bis-tris NuPAGE gel (Invitrogen NP0321BOX) at 150 V in 1X NuPAGE MOPS buffer (Invitrogen Cat#NP0001-02). The protein:RNA complexes were transferred to Hybond-N+ nitrocellulose membranes (GE Healthcare Cat#RPN303B) with NuPAGE transfer buffer (Invitrogen Cat#NP0006-1) for 1.5 h at 100V.

Labeled RNA was detected by autoradiography. The appropriate region was excised from the membrane and treated with 0.25 μg/μL Proteinase K (Roche Cat#03115836001) (50 mM Tris-HCl pH 7.5, 50 mM NaCl, 0.1% NP-40, 10 mM imidazole, 1% SDS, 5 mM EDTA, and 5 mM β-mercaptoethanol; 2 hr 55°C with shaking) in a 500 μL reaction. The RNA component was isolated with a standard phenol:chloroform extraction followed by ethanol precipitation. The RNA was reverse transcribed using Superscript III (Invitrogen Cat#18080-044) and the miRCat-33 RT oligo (IDT) for 1 hr at 50°C in a 20 μL reaction. The resulting cDNA was amplified by PCR in five separate reactions using La Taq (Takara, Cat#RR002M) (2 μL template, 18-21 cycles) PCR reactions were combined, precipitated in ethanol, and resolved on a 3% Metaphore agarose gel (Lonza Cat#50180). A region corresponding to 140 to 200 bp was excised from the gel and extracted using the Min-elute kit (QIAGEN Cat#28606). Libraries were sequenced by the Wellcome Trust Clinical Research Facility (Edinburgh, UK) on Next-Seq with single-end, 75nt output.

### Quantification and Statistical Analysis

#### Mass spectrometry analysis

The MaxQuant software platform (Cox and Mann, 2008) version 1.6.1.0 was used to process the raw files and search was conducted against *Saccharomyces cerevisiae* complete/reference proteome set of UniProt database (released on 14/06/2019), using the Andromeda search engine (Cox et al., 2011). For the first search, peptide tolerance was set to 20 ppm while for the main search peptide tolerance was set to 4.5 pm. Isotope mass tolerance was 2 ppm and maximum charge to 7. Digestion mode was set to specific with trypsin allowing maximum of two missed cleavages. Carbamidomethylation of cysteine was set as fixed modification. Oxidation of methionine and acetylation of the N-terminal were set as variable modifications. Multiplicity was set to 2 and for heavy labels Arginine 6 and Lysine 6 were selected. Peptide and protein identifications were filtered to 1% FDR. Only proteins identified with high confidence (peptides ≥ 2) were considered for further analysis.

For glucose starvation, we performed each time course in quadruplicate, and for heat shock in triplicate. The exception was the two-minute time point for each condition, which was only performed in duplicate. For each analysis, only proteins expressed in at least two replicates for every time point shown therein were included.

#### PCA

For the PCA analysis in Figure 1F, all TRAPP datasets were included. The raw log2 values were mainly between −2 and +2, and data normalization was not required. For each protein, the median value between replicates was used. In total, 302 proteins were present in at least two biological replicates for each time point and condition, and thus included in the analysis. For the PCA in Figure 1G, only the 16 min time points from glucose starvation, heat shock, and mock shift were included. PCA was calculated with python v3.6.8 using the Jupiter notebook and the scikit-learn v0.22.2 library.

#### Ribosome structure

The ribosome structure PDB: 3J77 (Figure 2) is derived from Svidritskiy et al. (2014). The mRNA was modeled into the structure, using PDB: 3J81 (Hussain et al., 2014).

#### CRAC analysis

The datasets were dumultiplexed using pyBarcodeFilter from the pyCRAC package (Webb et al., 2014). Flexbar v3.4.0 (Dodt et al., 2012) was used to remove sequencing adapters, trim low-quality positions from the 3′ end, and remove low-quality reads (parameters: -ao 4 -u 2 -q TAIL -m 11 -at RIGHT with adaptor sequence TGGAATTCTCGGGTGCCAAGGC). In addition to the barcode, each read contained three random nucleotides at the 5′ end to allow PCR duplicates to be removed by collapsing identical sequences with pyFastqDuplicateRemover (Webb et al., 2014). Reads were filtered to exclude low-entropy sequences using bbduk (https://sourceforge.net/projects/bbmap/) with parameters entropy = 0.5 entropywindow = 10 entropyk = 6. Because translation initiation factors largely target spliced mRNAs, the sequencing reads were mapped to a modified version of the *S. cerevisiae* EF4.74 genome (Ensembl) in which the introns had been bioinformatically removed. The reads were aligned using Novoalign v2.07.00, with reads mapping to multiple locations randomly assigned (-r Random).

The numbers of reads mapping to different mRNAs were determined using pyReadCounters (Webb et al., 2014) and a custom genome annotation file. Binding to individual mRNAs was quantified in one of two ways: 1) ‘total binding’, reflecting the number of reads mapping anywhere within a given transcript, and 2) ‘5′ end binding’, reflecting the number of reads mapping within the 5′ UTR and the first 150 nt of coding sequence. Three pseudocounts were added to each transcript to improve the quantification. Most analyses (Figures 4B–4E, S4D, and S4E) were based on the list of 2,000 transcripts showing the strongest binding to eIF4B, defined by the reads per million average between the ‘control’ and ‘mock shift’ conditions. In order to visualize binding across individual transcripts (e.g., Figure 4F), the coverage at each position along the genome was calculated and normalized to the library size using genomecov from bedtools v2.27.0 (Quinlan and Hall, 2010). The Integrative Genomics Browser was used to visualize binding across individual transcripts (Robinson et al., 2011). Coverage around start codons (Figures 4E, S4D, and S4E) was calculated using pyBinCollector (Webb et al., 2014). The resulting metaplots were generated in GraphPad Prism 8, while the heatmaps were made using Excel. Statistical analysis was performed using Graphpad. For Figures 4B and 4C, an unpaired t test was used to calculate changes in RNA binding before and after stress (for details, see the figure legend).

#### RNAseq data analysis

RNAseq reads were aligned to a concatenated genome consisting of the intronless *S. cerevisiae* genome and the 972H *Schizosaccharomyces pombe* genome using Novoalign. The reads mapping to the *S. pombe* genome were tabulated using pyReadCounters together with a custom annotation file. These values were used as a normalization standard to allow for quantitative comparisons between datasets. Genome coverage files (e.g., Figure 4F) were generated using genomecov from bedtools and scaled using values determined from the *S. pombe* spike-in control. For all analyses, we used the top 5,000 transcripts, defined by their average expression across all three conditions (control, glucose withdrawal 16 min, and heat shock 16 min). GO analysis was performed using GOrilla (Eden et al., 2009) on the 500 genes showing the greatest decline in mRNA levels. Violin plots were generated using GraphPad Prism 8.

## Supplemental Information

Document S1. Figures S1–S6

Table S1. DNA Oligonucleotides Used in This Study, Related to STAR Methods

Table S2. Global Changes in RNA Binding Determined by TRAPP, Related to Figure 1 Each value indicates the log2 fold change in RNA binding during stress compared to the control. The headings for each dataset indicate the strain (e.g., wt), the stress condition, the time point, the replicate number, and a unique identifier associated with each sample.

Table S3. Changes in Total Protein Abundance after 16 min of Glucose Starvation or Heat Shock, Related to Figure 1 Each value indicates the log2 fold change in RNA binding during stress relative to the control.

Table S4. eIF4A CRAC Results Showing the Amount of Binding (in Reads per Million) to Different mRNAs, Related to Figure 4

Table S5. eIF4B CRAC Results Showing the Amount of Binding (in Reads per Million) to Different mRNAs, Related to Figure 4

Table S6. Ded1 CRAC Results Showing the Amount of Binding (in Reads per Million) to Different mRNAs, Related to Figure 4

Table S7. Heatmaps Corresponding to Figure S4E, Related to Figure 4

Table S8. Global Changes in RNA Levels during Stress, Related to Figure 5 Values show reads per kilobase and were normalized to the *S. pombe* spike-in control.

Document S2. Article plus Supplemental Information
