# Supplementary material for: Stress-Induced Translation Inhibition through Rapid Displacement of Scanning Initiation Factors
Source: Mol Cell. 2020 Nov 5;80(3):470–484.e8. doi: 10.1016/j.molcel.2020.09.021 (PMC7657445; doi:10.1016/j.molcel.2020.09.021)

Molecular Cell, Volume *80*

Supplemental Information

Stress-Induced Translation Inhibition through Rapid Displacement of Scanning Initiation Factors

Stefan Bresson, Vadim Shchepachev, Christos Spanos, Tomasz W. Turowski, Juri Rappsilber, and David Tollervey

SUPPLEMENTARY FIGURE LEGENDS

Figure S1. The impact of stress on the yeast RNA binding proteome. Related to Figure 1.
(A) Bar chart showing all proteins with greater than 2-fold change in RNA association after 16 min of heat shock *(left)* or a mock shift *(center)* in TRAPP analyses*.* The right-hand panel shows changes in protein abundance following 16 min of heat shock *(right).* (B) Global changes in protein abundance following glucose starvation or heat shock. RNA binding proteins (RBPs) are highlighted in pink. (C) Comparison of changes in RNA association for individual RBPs after 2 min of glucose starvation, versus treatment for 2 min with 2 mM sorbate at pH 5. (D) Time course showing changes in RNA association for selected proteins.

Figure S2. Changes in RNA binding among translation initiation factors. Related to Figure 3.
(A) Polysome gradient analyses from control, untreated cells (*left*) and following glucose withdrawal (*middle*) or heat shock at 42°C (*right*), for 16 min.
(B) Time course showing changes in RNA binding for all translation initiation factors following glucose starvation (blue) and heat shock (pink) from TRAPP analyses.

Figure S3. Genome-wide analysis of the RNA binding profiles of eIF4A, eIF4B, and Ded1. Related to Figure 4.
(A) Representative autoradiogram of protein:RNA complexes separated by SDS-PAGE during the CRAC analyses. (B) The total number of reads in each CRAC dataset. Reads from separate replicates were merged together. (C) Breakdown of Ded1- and eIF4A-bound RNAs by biotype. (D) Metaplots showing the distribution of eIF4A, eIF4B, and Ded1 binding around the mRNA start codon for individual replicates. The number of replicates is shown in the upper right corner. (E) Heatmaps showing the distribution of eIF4B binding around the mRNA start codon, sorted by decreasing binding. Scales are shown at the bottom. The upper part of each heatmap is scaled differently (0-1000) due to the very high number of read mapping to those transcripts. Regions that exceed the scale are colored black.

Figure S4. Binding of eIF4A, eIF4B, and Ded1 to ribosomal RNA. Related to Figure 4.
(A) CRAC analysis showing binding of eIF4A, eIF4B, and Ded1 across the 35S pre-rRNA. (C) A close-up view of the mRNA exit channel showing the crosslinking sites on 18S for Ded1 (green) and eIF4B (teal).

Figure S5. Binding of eIF4A, eIF4B, and Ded1 to specific transcripts. Related to Figure 4.
(A) CRAC analysis showing binding of eIF4A, eIF4B, and Ded1 across a number of selected mRNAs. Each set of tracks is normalized to total library size using reads per million, with the exact value indicated in the upper right corner of each box. RNAseq traces are shown at the bottom as a control. Each track is normalized to a spike-in control, and thus represents the absolute abundance of each mRNA compared to the control. (B) Heatmaps showing the distribution of Ded1 around the mRNA start codon. Transcripts are sorted by the ratio of 5′ binding (5' UTR to +150 from start codon) versus downstream binding in the control sample for each species. Only transcripts longer than 700 nt were included in the analysis (n = 1,654). (C) Same as (B) but for eIF4B. (D) Boxplot quantifying the ratio of 3' binding to 5' binding for eIF4B and Ded1. (E) Scatterplot comparing Ded1 binding to mRNA levels. The *DED1* transcript itself is highlighted in teal.

Figure S6. **Comparisons of datasets. Related to Figure 5.**
(A) Comparison of replicate RNAseq datasets. Pearson correlations for RNAseq data replicates for all mRNAs for which sequence reads were recovered (n=6,273).
(B) Comparison of stress-induced changes in 5′-end binding of the indicated translation factors at 16 minutes, as determined by CRAC (Y axis), relative to changes in mRNA abundance, as determined by RNAseq with a spike-in control (X axis). The top 2,000 transcripts are shown for eIF4B and Ded1, whereas the top 500 are shown for eIF4A, due to reduced read coverage. Upper panels; glucose withdrawal. Lower panels; heat shock. Points below the dotted red line indicate mRNAs for which the reduction in factor binding was greater than the reduction in mRNA abundance.

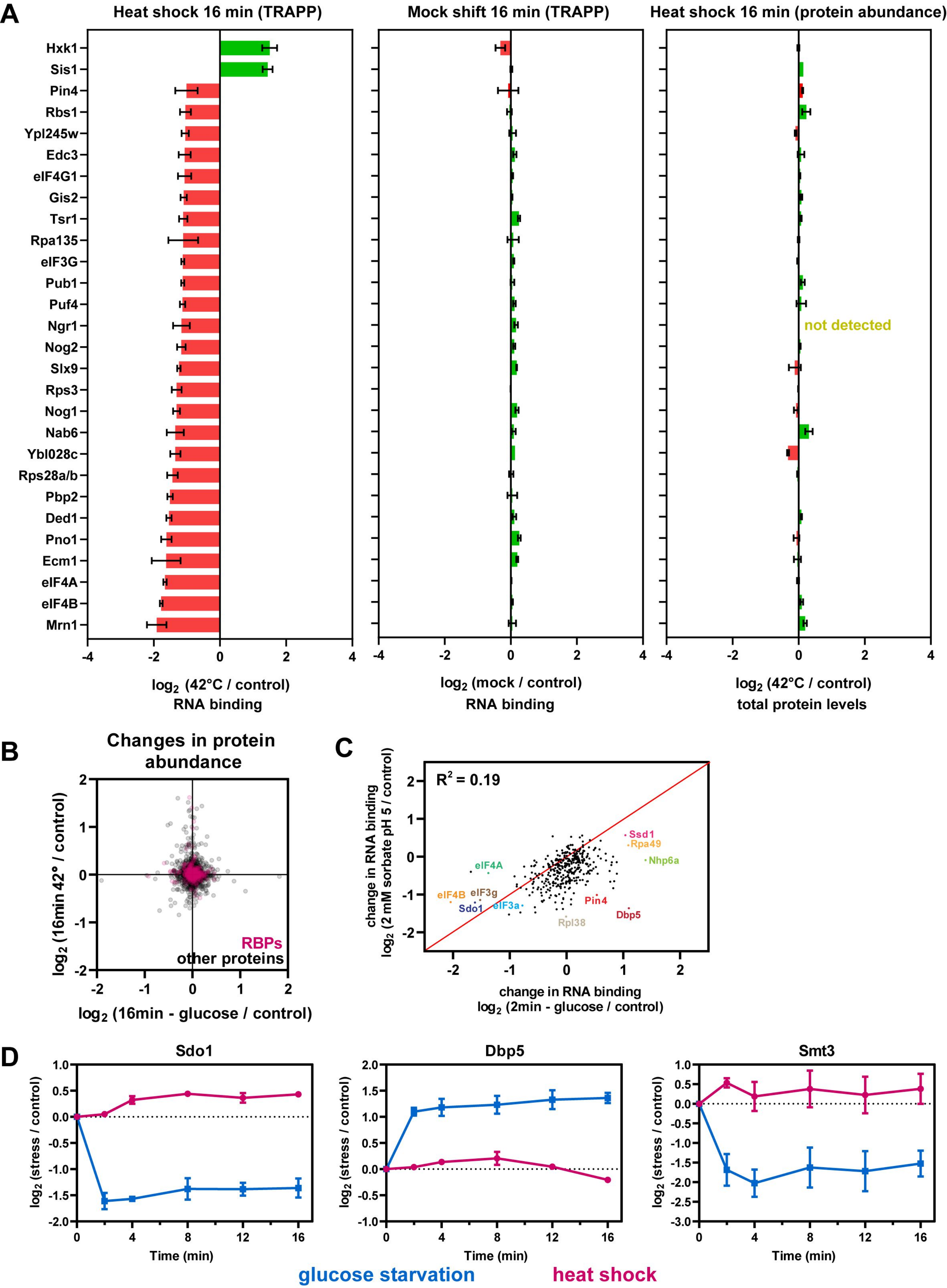

Bresson et al. Fig S1

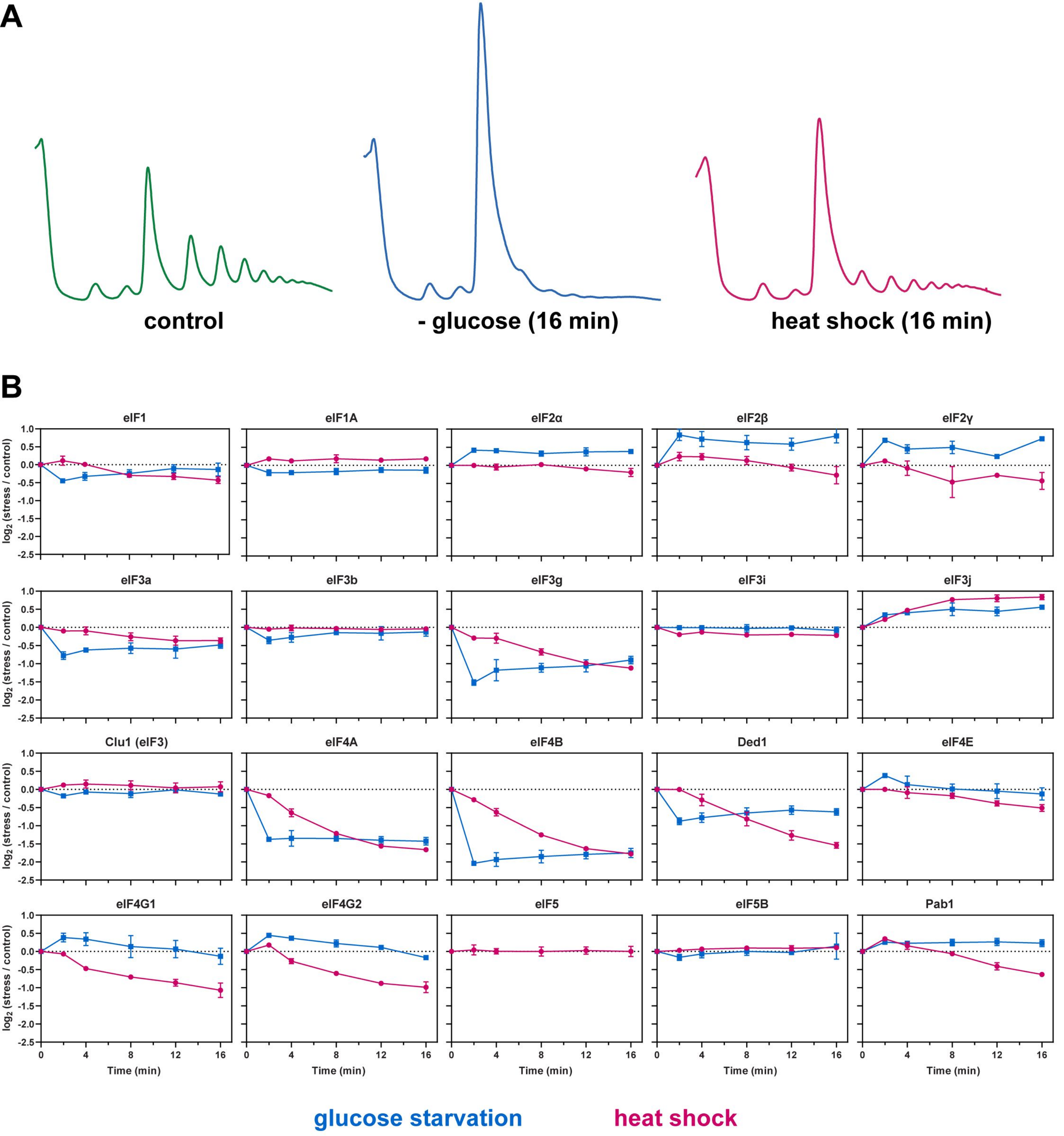

Bresson et al. Fig. S2

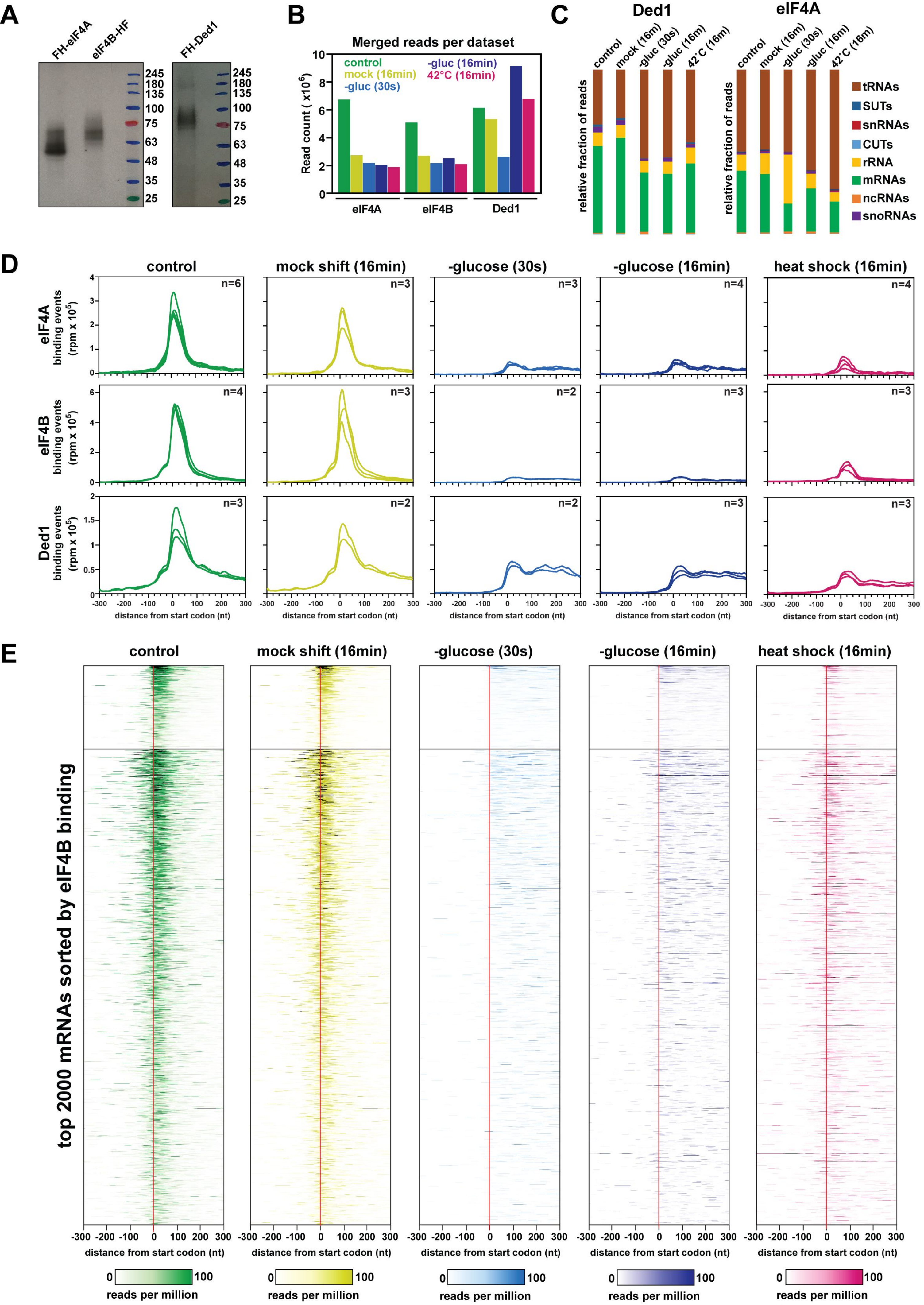

Bresson et al. Fig. S3

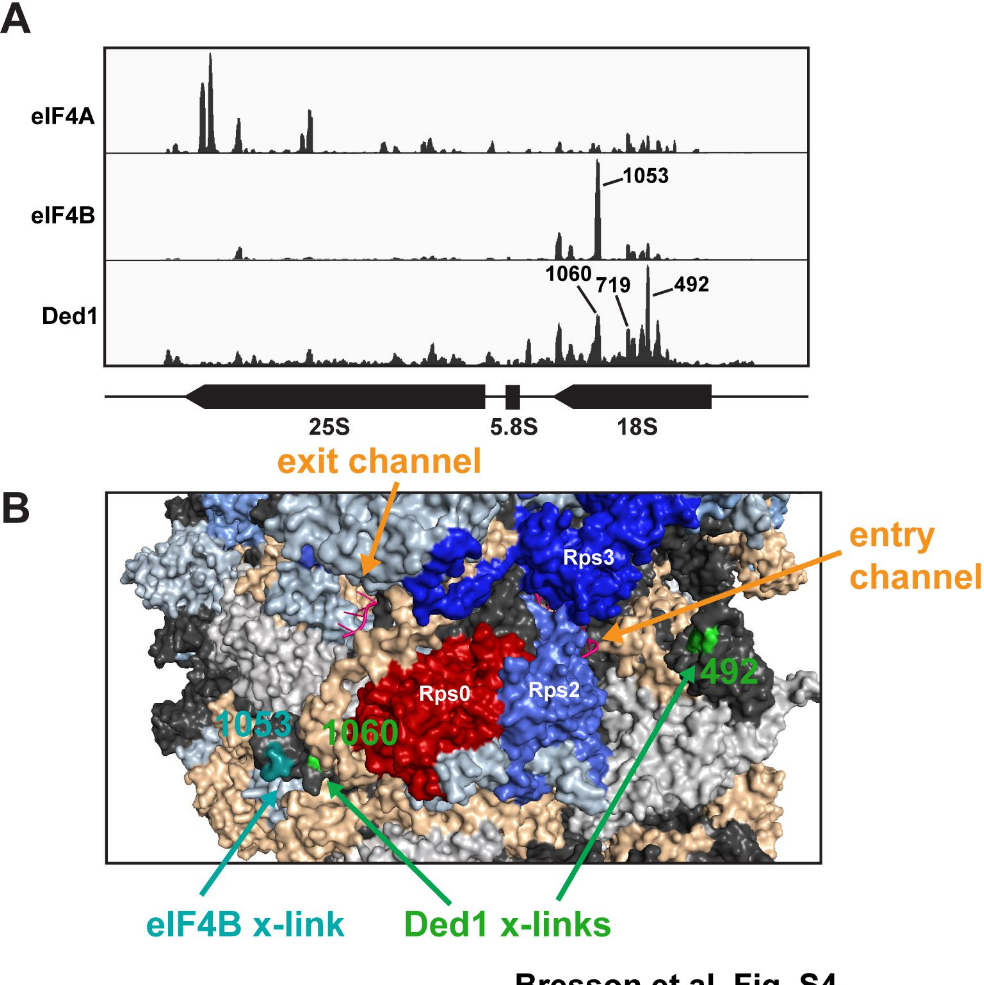

Bresson et al. Fig. S4

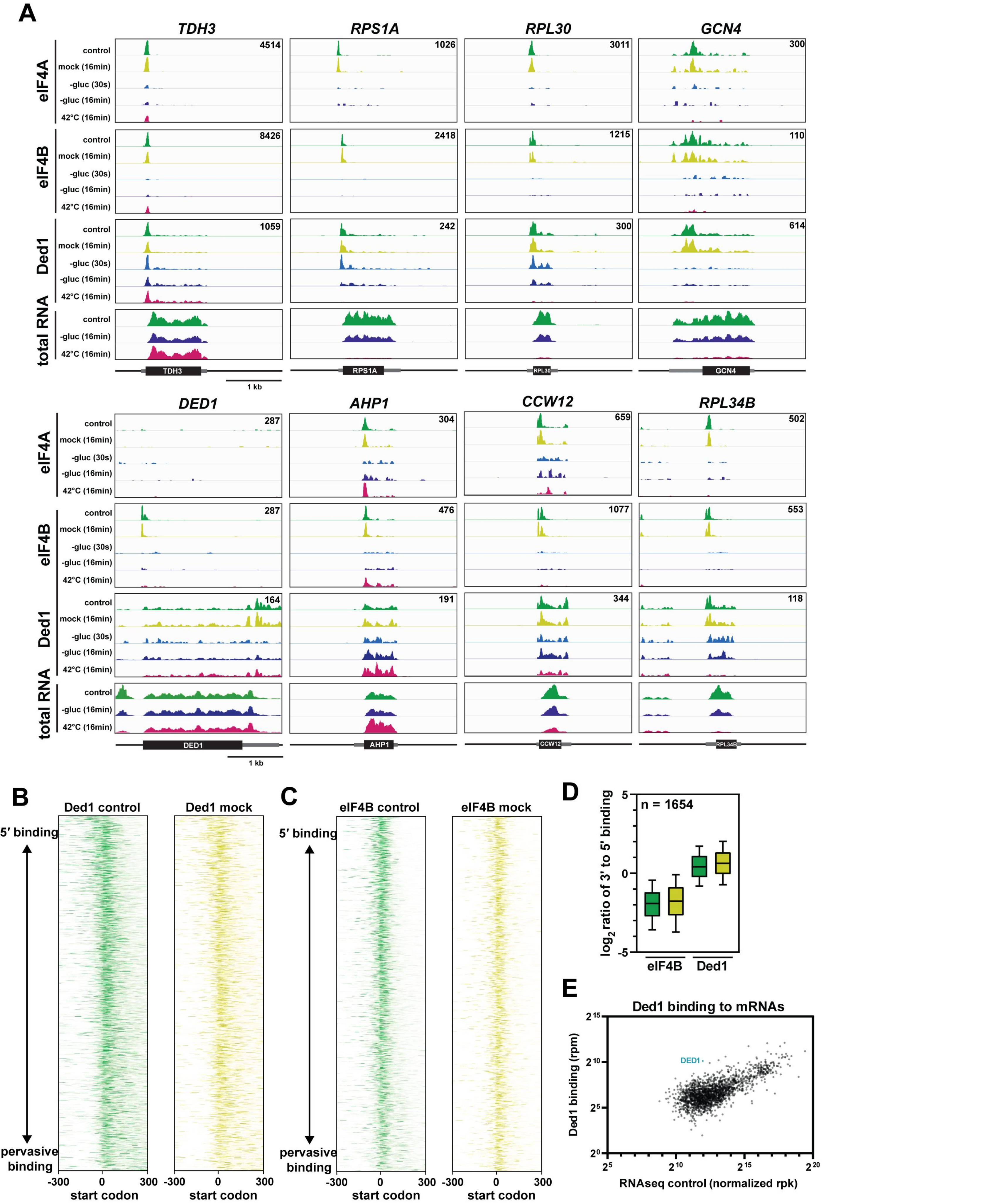

Bresson et al. Fig S5

A

			wild type control			wild type -glucose		wild type 42˚C			wild type 42˚C + chx		Δski2 control		Δski2 42˚C		Δxrn1 control		Δxrn1 42˚C	
wild type	control		1	0.9997	0.9933	0.8777	0.8782	0.4351	0.4512	0.3857	0.4067	0.4847	0.9964	0.9938	0.3666	0.4191	0.9095	0.9526	0.4014	0.4515
			0.9997	1	0.9949	0.875	0.8761	0.4299	0.4458	0.3811	0.4047	0.4827	0.9969	0.9944	0.363	0.4148	0.9152	0.9547	0.3995	0.4489
			0.9933	0.9949	1	0.8659	0.8689	0.4204	0.4362	0.3739	0.4035	0.4803	0.9971	0.9935	0.3593	0.409	0.9351	0.9618	0.3997	0.4469
	-glucose		0.8777	0.875	0.8659	1	0.999	0.6771	0.6842	0.644	0.6697	0.7377	0.8777	0.8697	0.6316	0.6798	0.7802	0.8417	0.6791	0.7218
			0.8782	0.8761	0.8689	0.999	1	0.6743	0.6816	0.6416	0.6683	0.7364	0.8794	0.8703	0.6305	0.6773	0.7873	0.8439	0.6791	0.7209
	42˚C		0.4351	0.4299	0.4204	0.6771	0.6743	1	0.9963	0.9881	0.898	0.8991	0.4406	0.4301	0.978	0.9806	0.357	0.4105	0.9039	0.9119
			0.4512	0.4458	0.4362	0.6842	0.6816	0.9963	1	0.9745	0.8749	0.878	0.4567	0.4451	0.9649	0.9677	0.3695	0.4249	0.8818	0.8906
			0.3857	0.3811	0.3739	0.644	0.6416	0.9881	0.9745	1	0.9312	0.923	0.3928	0.3828	0.9918	0.9885	0.3236	0.3693	0.9296	0.9346
	42˚C + chx		0.4067	0.4047	0.4035	0.6697	0.6683	0.898	0.8749	0.9312	1	0.9917	0.4176	0.408	0.9299	0.9239	0.3979	0.4252	0.9321	0.9389
			0.4847	0.4827	0.4803	0.7377	0.7364	0.8991	0.878	0.923	0.9917	1	0.4946	0.4866	0.9211	0.9238	0.4619	0.495	0.9338	0.9452
Δski2	control		0.9964	0.9969	0.9971	0.8777	0.8794	0.4406	0.4567	0.3928	0.4176	0.4946	1	0.9951	0.3762	0.427	0.9236	0.9603	0.4137	0.462
			0.9938	0.9944	0.9935	0.8697	0.8703	0.4301	0.4451	0.3828	0.408	0.4866	0.9951	1	0.3653	0.4183	0.9183	0.9564	0.4033	0.4535
	42˚C		0.3666	0.363	0.3593	0.6316	0.6305	0.978	0.9649	0.9918	0.9299	0.9211	0.3762	0.3653	1	0.9924	0.328	0.364	0.9501	0.946
			0.4191	0.4148	0.409	0.6798	0.6773	0.9806	0.9677	0.9885	0.9239	0.9238	0.427	0.4183	0.9924	1	0.3637	0.408	0.9551	0.9578
Δxrn1	control		0.9095	0.9152	0.9351	0.7802	0.7873	0.357	0.3695	0.3236	0.3979	0.4619	0.9236	0.9183	0.328	0.3637	1	0.9803	0.41	0.4438
			0.9526	0.9547	0.9618	0.8417	0.8439	0.4105	0.4249	0.3693	0.4252	0.495	0.9603	0.9564	0.364	0.408	0.9803	1	0.4383	0.4806
	42˚C		0.4014	0.3995	0.3997	0.6791	0.6791	0.9039	0.8818	0.9296	0.9321	0.9338	0.4137	0.4033	0.9501	0.9551	0.41	0.4383	1	0.9949
			0.4515	0.4489	0.4469	0.7218	0.7209	0.9119	0.8906	0.9346	0.9389	0.9452	0.462	0.4535	0.946	0.9578	0.4438	0.4806	0.9949	1

Pearson correlation

0 0.5 1

B

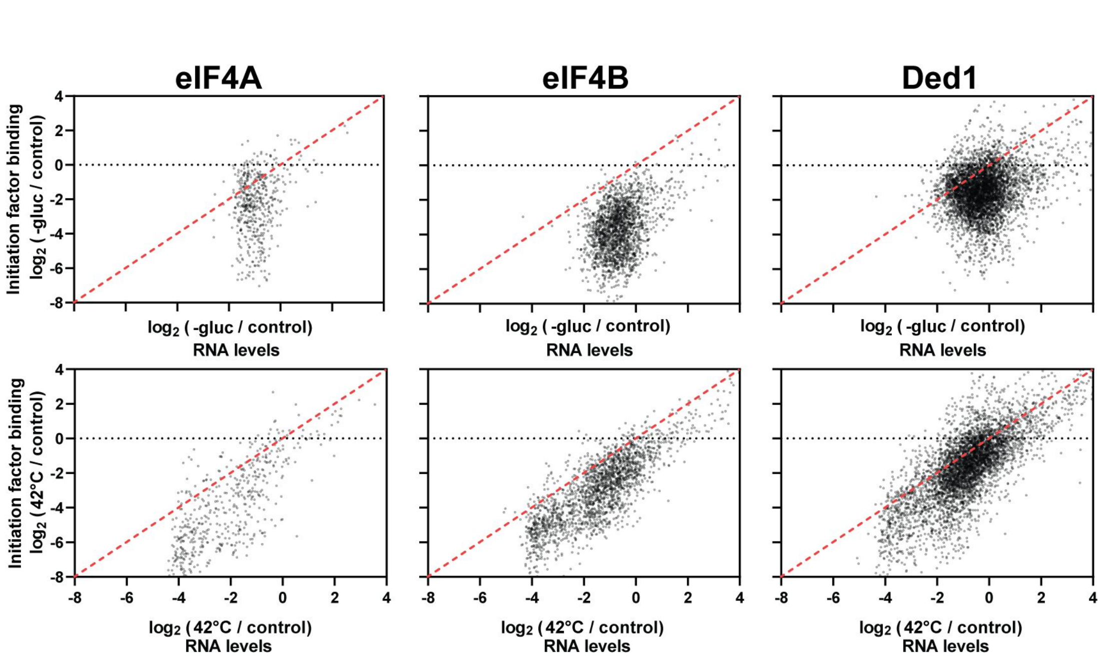

Supplement: Document S1. Figures S1–S6 [file mmc1.pdf]
